# Trajectory of response to esketamine nasal spray for treatment resistant depression: Findings from ESCAPE-TRD

**DOI:** 10.1192/j.eurpsy.2026.12229

**Published:** 2026-06-16

**Authors:** Allan H. Young, Lucie Bartova, Narcís Cardoner, Koen Demyttenaere, Andrea Fagiolini, Yordan Godinov, José Felipe Golib Dzib, Christian von Holt, Roger S. McIntyre, Albino J. Oliveira-Maia, Benoit Rive, Philip Gorwood

**Affiliations:** 1 Institute of Psychiatry, Psychology and Neuroscience, King’s College London, Department of Psychological Medicine, London, UK; 2 South London and Maudsley NHS Foundation Trust, Bethlem Royal Hospital, Beckenham, UK; 3 Division of Psychiatry, Department of Brain Sciences, Faculty of Medicine, Imperial College London, London, UK; 4 Department of Psychiatry and Psychotherapy, Medical University of Vienna, Vienna, Austria; 5 Comprehensive Center for Clinical Neurosciences and Mental Health, Medical University of Vienna, Vienna, Austria; 6 Institut d’Investigació Biomèdica Sant Pau, Hospital de la Santa Creu i Sant Pau, Barcelona, Spain; 7 CIBERSAM, Carlos III Health Institute, Madrid, Spain; 8 Universitat Autònoma de Barcelona, Barcelona, Spain; 9 University Psychiatric Centre KU Leuven, Campus Leuven, Belgium; 10 Department of Molecular Medicine, University of Siena School of Medicine, Siena, Italy; 11 Johnson & Johnson, Sofia, Bulgaria; 12 Johnson & Johnson, Madrid, Spain; 13 Johnson & Johnson, Neuss, Germany; 14 Department of Psychiatry, University of Toronto, Toronto, ON, Canada; 15 Department of Pharmacology and Toxicology, University of Toronto, ON, Canada; 16 Champalimaud Research and Clinical Centre, Champalimaud Foundation, Lisbon, Portugal; 17 NOVA Medical School, Universidade NOVA de Lisboa, Lisbon, Portugal; 18 Johnson & Johnson, Paris, France; 19 Université Paris Cité, Institute of Psychiatry and Neuroscience of Paris (IPNP), INSERM U1266, Paris, France; 20 GHU Paris Psychiatrie et Neurosciences (CMME), Hôpital Sainte-Anne, Paris, France.

**Keywords:** esketamine, long-term outcomes, trajectory of response, treatment resistant depression

## Abstract

**Background:**

Previous research has demonstrated the positive impact of early remission on disease trajectory for patients with major depressive disorder. However, there is a paucity of literature assessing the response trajectory for patients with treatment resistant depression (TRD). These analyses investigated patient outcome trajectories to Week 32, including the relationship between short- and long-term outcomes with esketamine nasal spray (ESK-NS) treatment and symptom resolution.

**Methods:**

ESCAPE-TRD (NCT04338321), a randomized, open-label, phase IIIb study, evaluated the efficacy and safety of ESK-NS versus quetiapine extended release (QTP-XR) in patients with TRD. Rater-blinded Montgomery-Åsberg Depression Rating Scale (MADRS) total score was used to assess depressive symptoms and the trajectory of patient responses to Week 32. For ESK-NS versus QTP-XR, time to symptom resolution across MADRS items was evaluated using Kaplan–Meier analyses.

**Results:**

Of 676 patients, 336 and 340 were randomized to ESK-NS and QTP-XR, respectively. Most ESK-NS-treated patients achieved continuously improved outcomes to Week 32; early response was associated with better long-term outcomes. Among patients who achieved Week 4 response, 89.6% achieved remission at any point from Week 4 to 32; 78.3% of Week 8 responders achieved remission at any point from Week 8 to 32. Greater and earlier symptom resolution was observed with ESK-NS versus QTP-XR across most MADRS items.

**Conclusions:**

Most ESK-NS-treated patients with TRD showed continuous improvements over time, with short-term response associated with better long-term outcomes. Long-term improvements were observed even among partial/minimal early responders, reinforcing the value of longer treatment. ESK-NS had a positive impact across the full spectrum of MADRS-assessed symptoms.

## Introduction

Major depressive disorder (MDD) has a profound negative impact on patients’ lives, imposing extensive mental and physical symptoms, including poor concentration, anhedonia, suicidality, disrupted sleep, and/or lack of appetite [1–5]. Anhedonia, a diminished interest or pleasure in all, or almost all, activities, has been associated with a particularly poor prognosis [1–3, 6, 7]. Substantial alleviation across the full spectrum of symptoms is an important treatment goal for patients, and subsequent treatment should focus on preventing relapse [8, 9].

Treatment resistant depression (TRD), commonly defined as a failure to respond to at least two consecutive antidepressant pharmacological treatments of adequate dose and duration within the same major depressive episode, affects ~10–30% of patients with MDD, with some estimates ranging up to 55% [10–15]. TRD is associated with a greater clinical burden than MDD, as patients are less likely to achieve remission and more likely to experience relapse [16]. Consequently, managing TRD often requires more complex treatment strategies; however, current clinical treatment pathways remain highly heterogeneous across settings [11, 17, 18].

Previous research in MDD has explored individual patient-level variability in response to antidepressant monotherapy, demonstrating the impact of early symptom improvements and remission in shaping disease trajectory [19, 20]. Despite limited or nonresponse, patients with TRD often remain on the same treatment for long periods, highlighting the need to understand how early clinical changes, or lack thereof, can inform future prognosis and support personalized care [21–23]. For example, the Montgomery-Åsberg Depression Rating Scale (MADRS) can be used to assess how early resolution of individual symptoms may facilitate improved overall outcomes for patients. A deeper understanding of how the trajectory of treatment response may predict future outcomes, and the potential benefits of continuing treatment despite limited early response, may aid personalized treatment decisions and increase the likelihood of favorable and sustained outcomes [21–23].

Esketamine nasal spray (NS), administered in combination with a selective serotonin reuptake inhibitor (SSRI) or serotonin norepinephrine reuptake inhibitor (SNRI), is approved in Europe for the treatment of TRD [24, 25]. ESCAPE-TRD demonstrated the superior efficacy of esketamine NS over quetiapine extended release (XR) in the treatment of TRD [26]. These secondary analyses investigated the trajectory of response to esketamine NS treatment and compared the time to symptom resolution between treatment groups in ESCAPE-TRD.

## Methods

### Study design and patients

ESCAPE-TRD (NCT04338321), a randomized, open-label, MADRS rater-blinded, active-controlled phase IIIb study, evaluated the efficacy and safety of esketamine NS versus quetiapine XR in combination with an ongoing SSRI/SNRI, in patients with TRD (Supplementary Figure S1). The full methodology was reported in the primary publication [26].

### Outcomes

#### Patient response levels over time

The MADRS was used to assess symptom severity across 10 items. Total MADRS scores range from 0 to 60, with higher scores indicating more severe depression. Here, remission was defined as MADRS total score ≤10. Other outcomes were considered “without remission” (MADRS total score >10); minimal response, partial response and response were defined as a <25%/25–<50%/≥50% reduction from baseline in MADRS, respectively. Outcomes were mutually exclusive, and patients were categorized by their highest outcome achieved at each visit while on treatment throughout the study. Proportions of patients who reached these outcomes with esketamine NS and quetiapine XR were computed. Additionally, the proportion of patients “off study treatment” at each visit is reported for both treatment groups. Patient-level transitions between outcomes are reported at each visit through Week 32 for patients who received esketamine NS.

Stacked bar charts are presented to depict changes over time in the distribution of outcomes with esketamine NS versus quetiapine XR. At each time point, the height of each color-coded segment indicates the proportion of patients with the corresponding outcome. Furthermore, to illustrate patient-level transitions between outcomes across successive time points, alluvial diagrams (i.e., a specialized form of Sankey diagram) are presented. Patients’ transitions between outcomes are represented by linking bands between stacked bar charts, with the thickness of each linking band proportional to the number of patients in each transition.

#### Short-term and long-term outcomes

The relationship between short-term (Week 4/8) and long-term (response/remission at any point from Week 4 or Week 8) outcomes was assessed at Week 32 for patients who received esketamine NS. First, the proportions of patients who achieved response or remission at least once from Week 4 to 32 and from Week 8 to 32 were derived; these outcomes were not mutually exclusive, all patients who achieved remission are also reported as having achieved response. Second, transitions between outcomes at Week 4/8 and Week 32 were assessed, illustrating long-term outcomes based on patients’ short-term outcomes.

The stability of short-term outcomes was assessed over 32 weeks; among patients who achieved different outcomes at Week 4/8, the trajectory of individual patient outcomes at each subsequent visit is reported. In addition, the proportions of patients who achieved remission at a given week and remained in stable remission for each subsequent visit are reported to Week 32.

#### Time to event analysis (symptom-level comparison)

The prevalence of MADRS-defined items at baseline is reported for both treatment groups and the overall study population. Remission in MADRS (i.e., a score of 0–10 out of 60) was scaled down to individual items to assess time to symptom resolution, allowing symptom-level comparison between treatment groups. MADRS item scores were evaluated as dichotomous outcomes for patients with a baseline item score of ≥2. Over 32 weeks, symptom resolution was assessed; a score of 0–1 was defined as symptom resolution, with 0 indicating complete absence of the symptom and 1 demonstrating that the patient remained below the threshold level for symptom characterization. A score of 2–6 denoted the presence of the respective symptom (at various levels of severity). Resolution across each of the 10 MADRS items would result in a total MADRS score of ≤10, meeting the threshold for remission defined in these analyses.

### Statistical analysis

For patient trajectories per treatment arm analyses, data from on-treatment visits were utilized and the proportion of patients off study treatment is reported at each visit; for patients who remained on treatment, missing visits were imputed using last observation carried forward (LOCF). Patients who discontinued study treatment were categorized as “off study treatment” and could initiate alternative therapies; the association between short- and long-term outcomes was not assessed for patients who had discontinued treatment at either of the short-term time points (Week 4 or Week 8). In addition, the trajectory of a patient’s outcomes was not reported following discontinuation of study treatment at any point over 32 weeks.

Time to symptom resolution was estimated using Kaplan–Meier analyses for each MADRS item. Hazard ratios (HRs), with 95% confidence intervals (CIs), are also reported, using Cox proportional hazards models across MADRS items. Patients discontinuing study treatment before having experienced symptom resolution were censored at an infinite (arbitrarily large) time and assumed to never achieve the relevant event; patients completing treatment without symptom resolution were censored at the time of completion. All reported *p*-values are nominal and were not adjusted for multiple testing. A threshold for statistical significance of 5% was applied throughout.

For proportions of patients with resolution of symptoms based on MADRS, treatment discontinuations were imputed as negative outcomes (Supplementary Tables S1–S10; nonresponder imputation [NRI]; full methodology in Appendix 1).

## Results

### Patient disposition and baseline characteristics

Of 676 patients, 336 and 340 patients were randomized to esketamine NS and quetiapine XR, respectively [26]. Baseline characteristics were generally consistent between groups and representative of the patient population [26]. At baseline, the prevalence of MADRS-defined items was similar between groups; a high proportion of patients presented with the majority of symptoms (Table 1). The overall baseline prevalence of reduced appetite (59.9%; *n/N* = 404/675) and suicidal thoughts (27.7%; *n/N* = 187/675) was lower than for other symptoms (Table 1).

**Table 1.** Baseline MADRS-defined symptom prevalence Table 1. long description.

Item	Esketamine NS + SSRI/SNRI *N* = 336	Quetiapine XR + SSRI/SNRI *N* = 339	Overall *N* = 675
Apparent sadness	324/336 (96.4%)	331/339 (97.6%)	655/675 (97.0%)
Reported sadness	333/336 (99.1%)	337/339 (99.4%)	670/675 (99.3%)
Inner tension	313/336 (93.2%)	321/339 (94.7%)	634/675 (93.9%)
Reduced sleep	289/336 (86.0%)	297/339 (87.6%)	586/675 (86.8%)
Reduced appetite	205/336 (61.0%)	199/339 (58.7%)	404/675 (59.9%)
Concentration difficulties	329/336 (97.9%)	322/339 (95.0%)	651/675 (96.4%)
Lassitude	329/336 (97.9%)	322/339 (95.0%)	651/675 (96.4%)
Inability to feel	325/336 (96.7%)	323/339 (95.3%)	648/675 (96.0%)
Pessimistic thoughts	306/336 (91.1%)	298/339 (87.9%)	604/675 (89.5%)
Suicidal thoughts	94/336 (28.0%)	93/339 (27.4%)	187/675 (27.7%)

*Note*: Full analysis set in ESCAPE-TRD; *N* = 676; baseline MADRS data were missing for one patient. Baseline symptom prevalence was defined as a baseline score of ≥2 in the respective MADRS item. Abbreviations: MADRS, Montgomery-Åsberg Depression Rating Scale; NS, nasal spray; SNRI, serotonin norepinephrine reuptake inhibitor; SSRI, selective serotonin reuptake inhibitor; XR, extended release.

### Trajectories of response

**Figure 1.**
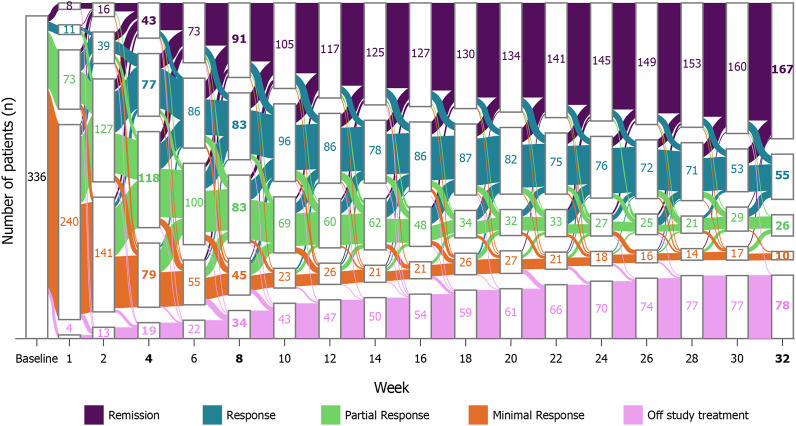
Patient trajectories with esketamine NS during ESCAPE-TRD (LOCF). Full analysis set in ESCAPE-TRD; *N* = 676; esketamine NS: *n* = 336. Patient trajectories between outcomes with esketamine NS treatment during ESCAPE-TRD. Remission was defined as MADRS total score ≤10. LOCF was applied for patients who remained on treatment with missing MADRS data at the given visit. Other outcomes were defined as follows, though all are considered “without remission” (MADRS total score >10): response was defined as a ≥50% reduction from baseline in MADRS, partial response as a 25–<50% reduction from baseline in MADRS, and minimal-response as a <25% reduction from baseline in MADRS. LOCF, last observation carried forward; NS, nasal spray; MADRS, Montgomery-Åsberg Depression Rating Scale. Figure 1. long description.

The achievement of improved outcomes fluctuated over time for individual patients treated with esketamine NS (Figure 1). At Week 8, 27.1% (*n/N* = 91/336) and 24.7% (*n/N* = 83/336) of patients who received esketamine NS achieved remission and response, respectively; a similar proportion achieved partial response at Week 8 (24.7%; *n/N* = 83/336).

The proportion of patients who responded positively to esketamine NS treatment continued to increase to Week 32: 49.7% (*n/N* = 167/336) and 16.4% (*n/N* = 55/336) of patients achieved remission and response at Week 32, respectively (Figure 1). At Week 32, the proportion of patients with minimal response (3.0%: *n/N* = 10/336) or partial response (7.7%: *n/N* = 26/336) decreased from the start of the study (Figure 1).

Patients who received quetiapine XR also demonstrated improvements in outcomes at Week 8 (Supplementary Figure S2). However, greater proportions of patients treated with esketamine NS achieved response or remission compared with quetiapine XR, as reported previously (Supplementary Figure S2) [26]. Over 32 weeks, patients treated with esketamine NS exhibited higher rates of remission and response compared with patients treated with quetiapine XR, and a greater proportion of patients who received quetiapine XR discontinued treatment.

### Short-term and long-term outcomes

**Figure 2.**
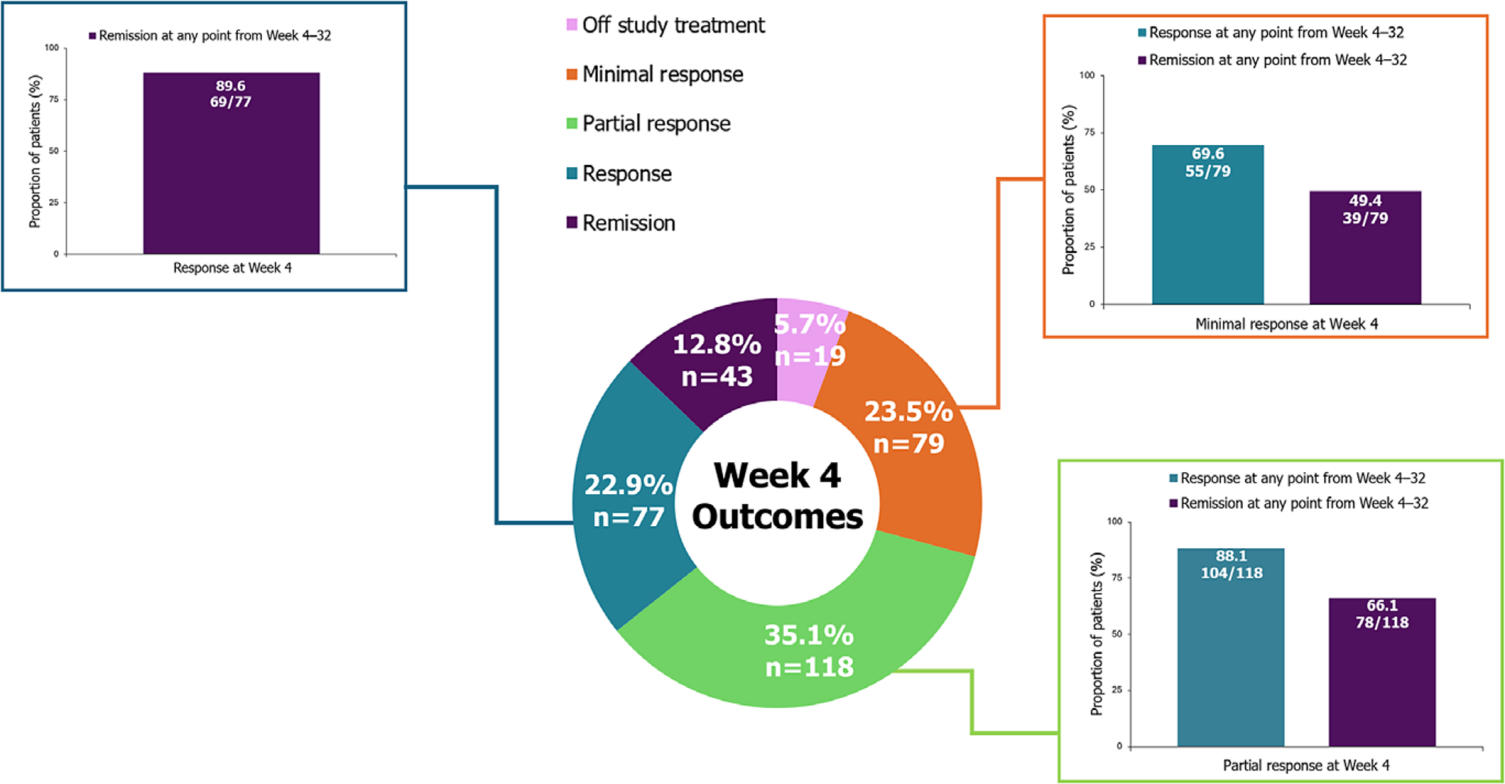
Short-term (Week 4) and long-term (any point from Week 4 to 32) outcomes in esketamine NS treatment. Full analysis set in ESCAPE-TRD; *N* = 676; esketamine NS: *n* = 336. For Week 4 outcomes, outcomes are reported as mutually exclusive, and “response” denotes patients who achieved response without remission (>10 in MADRS total score with ≥50% reduction in symptoms based on MADRS). By definition, 100% of patients who achieved response/remission at Week 4 also achieved response/remission at any point from Week 4 to 32. Outcomes for data reported between Week 4 and Week 32 are not mutually exclusive (all patients who achieved remission are also reported as having achieved response). LOCF was applied for patients who remained on treatment with missing MADRS data at Week 4. LOCF, last observation carried forward; MADRS, Montgomery-Åsberg Depression Rating Scale; NS, nasal spray. Figure 2. long description.

**Figure 3.**
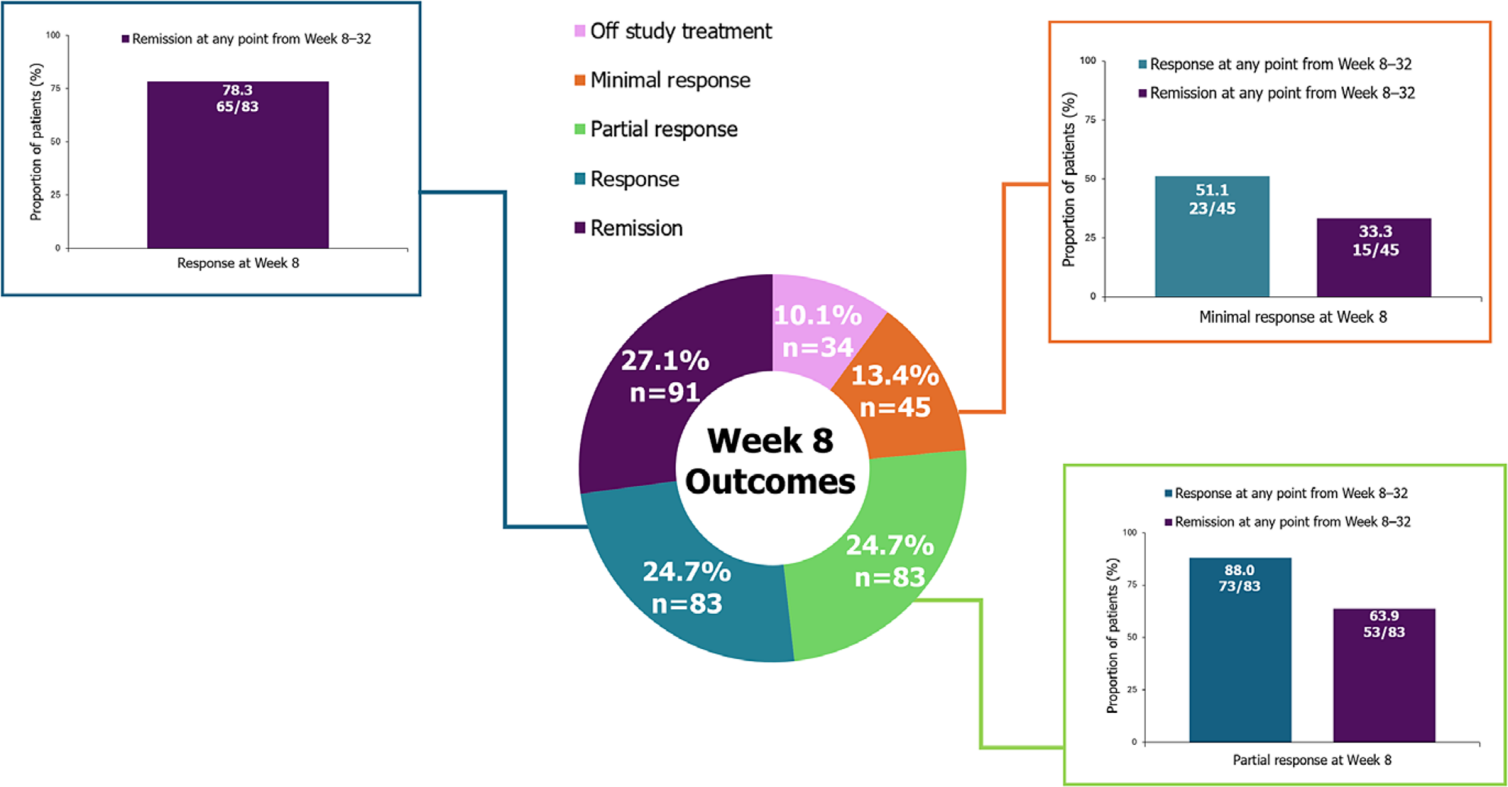
Short-term (Week 8) and long-term (any point from Week 8 to 32) outcomes in esketamine NS treatment. Full analysis set in ESCAPE-TRD; *N* = 676; esketamine NS: *n* = 336. For Week 8 outcomes, outcomes are reported as mutually exclusive, and “response” denotes patients who achieved response without remission (>10 in MADRS total score with ≥50% reduction in symptoms based on MADRS); by definition, 100% of patients who achieved response/remission at Week 8 also achieved response/remission at any point from Week 8 to 32. Outcomes for data reported between Week 8 and Week 32 are not mutually exclusive (all patients who achieved remission are also reported as having achieved response). LOCF was applied for patients who remained on treatment with missing MADRS data at Week 8. LOCF, last observation carried forward; MADRS, Montgomery-Åsberg Depression Rating Scale; NS: nasal spray. Figure 3. long description.

Among early responders to esketamine NS, high proportions demonstrated improved long-term outcomes. Of the patients who achieved response at Week 4, 89.6% (*n/N* = 69/77) proceeded to achieve remission at any point from Week 4 to 32; among Week 8 responders, 78.3% (*n/N* = 65/83) proceeded to achieve remission at any point from Week 8 to 32 (Figures 2 and 3). Moreover, among Week 4 partial responders, 66.1% (*n/N* = 78/118) and 88.1% (*n/N =* 104/118) proceeded to achieve remission and response at any point from Week 4 to 32, respectively (Figure 2). Similar results were observed for patients with partial response at Week 8 (Figure 3).

Among patients with minimal response to esketamine NS who remained on treatment at Week 4 (*n* = 79), improvements were observed over time as 69.6% (*n* = 55) achieved response and 49.4% (*n* = 39) achieved remission, at any point from Week 4 to 32 (Figure 2). Smaller proportions of Week 8 minimal responders (*n* = 45) achieved response (51.1%; *n* = 23) or remission (33.3%; *n* = 15) at any point from Week 8 to 32 (Figure 3).

**Figure 4.**
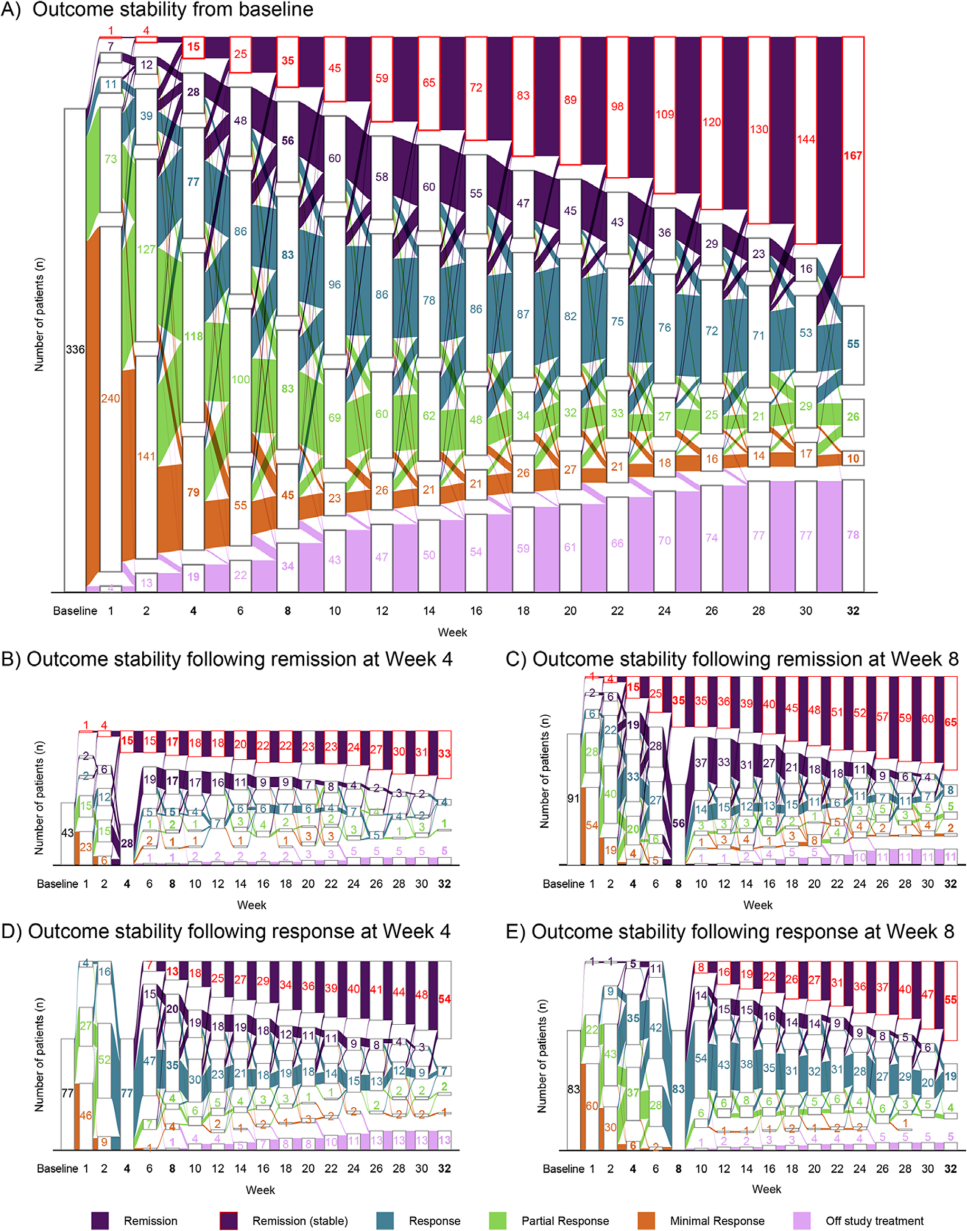
Outcome stability with esketamine NS treatment from baseline (A) and following remission at Week 4 (B), remission at Week 8 (C), response at Week 4 (D), and response at Week 8 (E) [LOCF]. Full analysis set in ESCAPE-TRD; *N* = 676; esketamine NS: *n* = 336. Patient trajectories between outcomes with esketamine NS treatment during ESCAPE-TRD. Remission was defined as MADRS total score ≤10. LOCF was applied for patients who remained on treatment with missing MADRS data at the given visit. Other outcomes were defined as follows, though all are considered “without remission” (MADRS total score >10): response was defined as a ≥50% reduction from baseline in MADRS, partial response as a 25–<50% reduction from baseline in MADRS, and minimal response as a <25% reduction from baseline in MADRS. Remission with stable outcome (highlighted in red) was defined for patients in remission (MADRS total score ≤10) at a given week who remained in remission for all subsequent weeks (using LOCF) until Week 32. LOCF, last observation carried forward; NS, nasal spray; MADRS, Montgomery-Åsberg Depression Rating Scale. Figure 4. long description.

The stability of outcomes achieved in the short term was assessed over 32 weeks (Figure 4A–E and Supplementary Figure S3A–D). From Week 1 to 32, continuous increases in the number of patients in stable remission were observed (Figure 4A). In general, higher proportions of patients who achieved response or remission at Week 4/8 proceeded to achieve stable remission compared with Week 4/8 partial or minimal responders (Figure 4B–E and Supplementary Figure S3A–D). Among patients who achieved response or remission at Week 4/8, high rates of response or remission were observed over 32 weeks (Figure 4B–E). Higher proportions of patients who achieved response, without reaching remission, at Week 4 proceeded to achieve remission at any point from Week 4 to 32 (89.6%; *n/N* = 69/77) compared with the proportions of patients who achieved response, without reaching remission, at Week 8 and proceeded to achieve remission at any point from Week 8 to 32 (78.3%; *n/N* = 65/83; Figures 2 and 3). Similarly, higher proportions of Week 4 partial responders achieved remission at any point from Week 4 to 32 (66.1%; *n/N* = 78/118) compared with the proportions of Week 8 partial responders who achieved remission at any point from Week 8 to 32 (63.9%; *n/N* = 53/83; Figures 2 and 3).

### Time to event analysis

**Figure 5.**
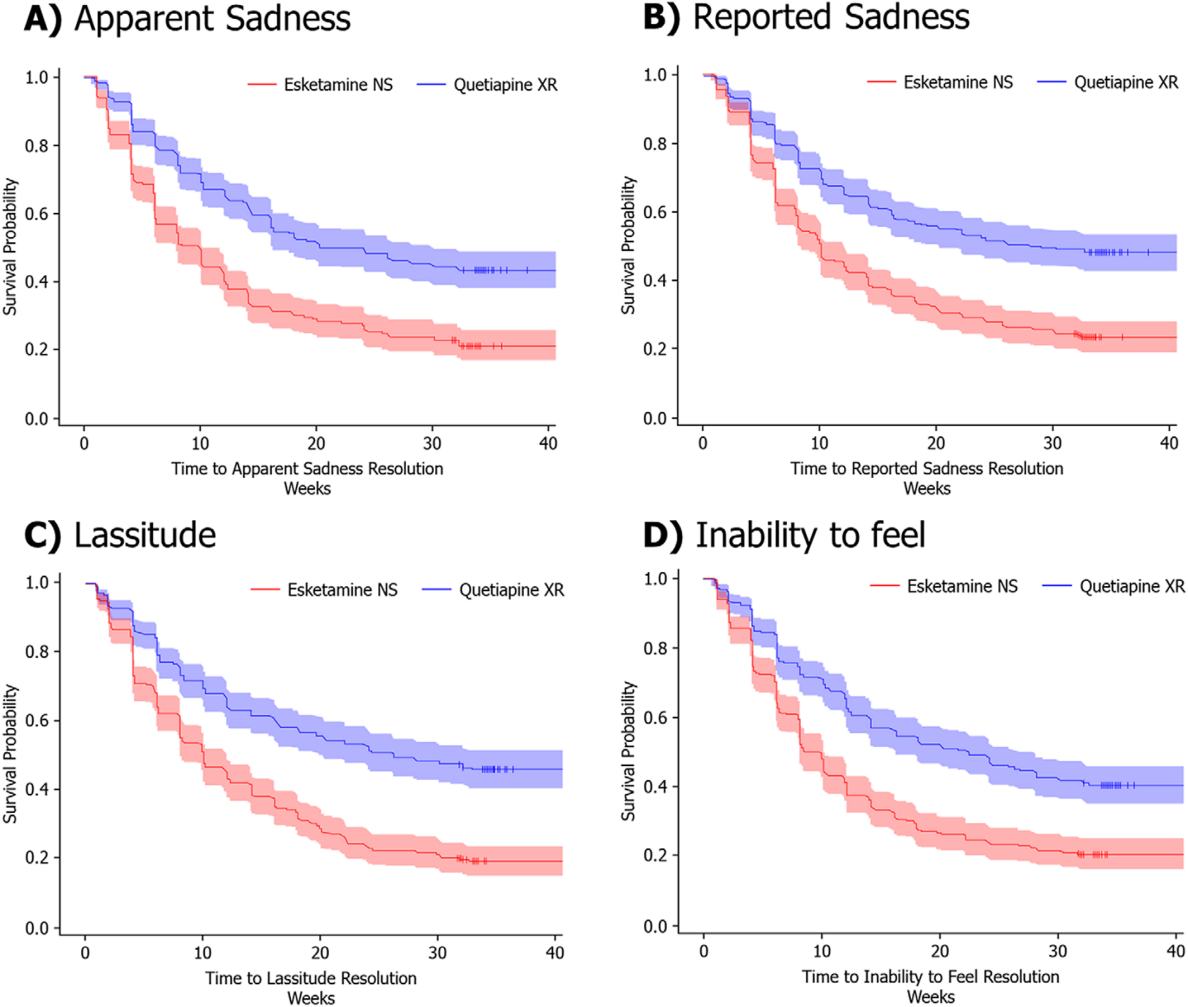
Time to symptom resolution for apparent sadness (A), reported sadness (B), lassitude (C), and inability to feel (D). Full analysis set in ESCAPE-TRD; MADRS item scores were evaluated as dichotomous outcomes for patients with a baseline item score of ≥2, “*n*” values are reported in Table 1; survival probabilities represent the estimated probability that the symptom is not resolved over time across items in MADRS, with 95% confidence intervals; MADRS, Montgomery-Åsberg Depression Rating Scale; NS, nasal spray; XR, extended release. Figure 5. long description.

**Figure 6.**
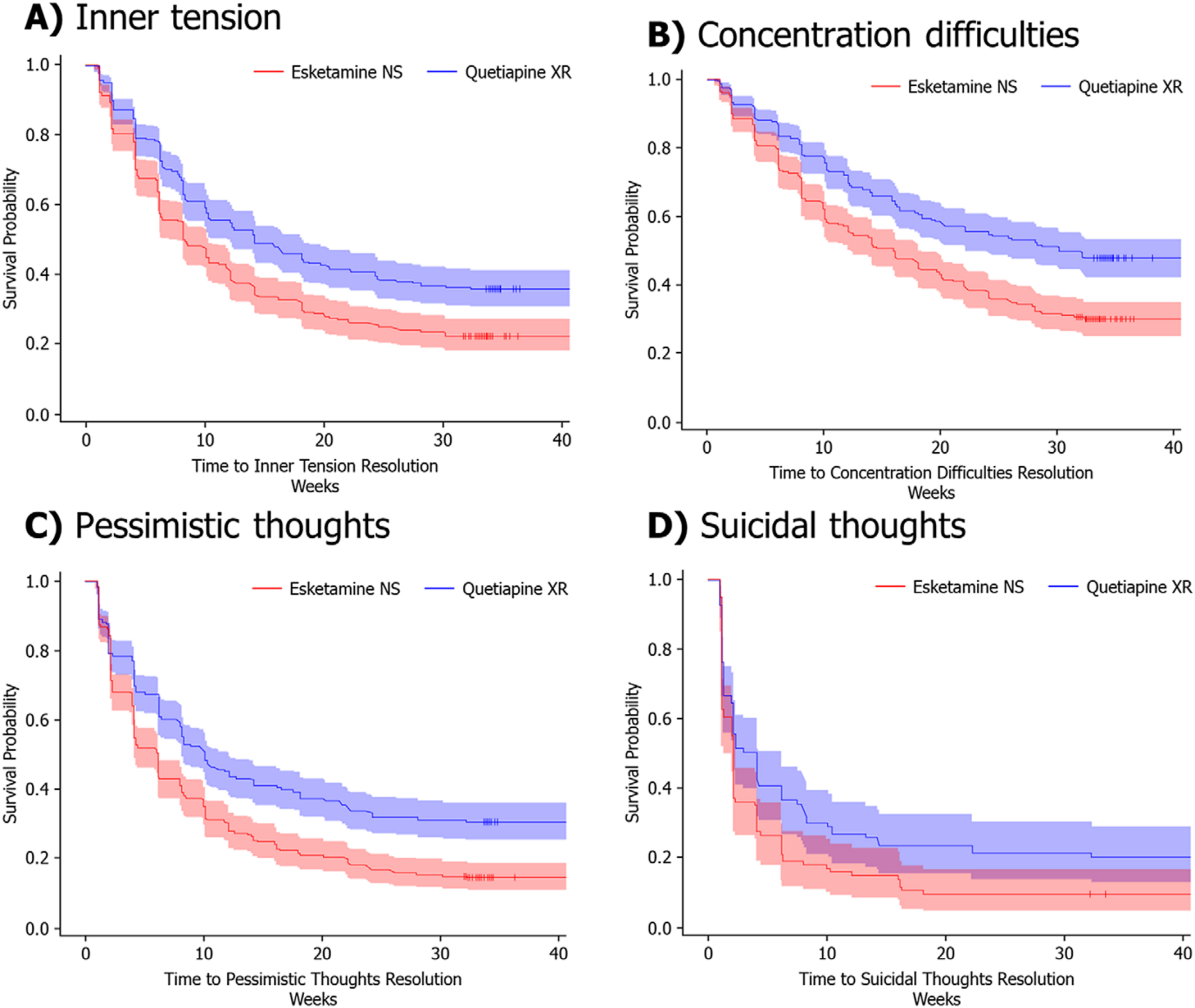
Time to symptom resolution for inner tension (A), concentration difficulties (B), pessimistic thoughts (C), and suicidal thoughts (D). Full analysis set in ESCAPE-TRD; MADRS item scores were evaluated as dichotomous outcomes for patients with a baseline item score of ≥2; “*n*” values are reported in Table 1. Survival probabilities represent the estimated probability that the symptom is not resolved over time across items in MADRS, with 95% confidence intervals; MADRS, Montgomery-Åsberg Depression Rating Scale; NS, nasal spray; XR, extended release. Figure 6. long description.

Symptom resolution consistently occurred earlier with esketamine NS versus quetiapine XR across the following symptoms: apparent sadness, reported sadness, lassitude, inability to feel, inner tension, concentration difficulties, pessimistic thoughts, and suicidal thoughts (Figures 5 and 6). HRs indicated a consistently higher probability of symptom resolution across most MADRS-defined items, with esketamine NS than with quetiapine XR (Table 2).

**Table 2.** Comparative hazard ratios for symptom resolution: Esketamine NS versus quetiapine XR Table 2. long description.

Item	Hazard ratio (95% CI), *p*-value
Apparent sadness	1.94 (1.61, 2.35), <0.0001
Reported sadness	1.96 (1.62, 2.38), <0.0001
Inner tension	1.49 (1.24, 1.79), <0.0001
Reduced sleep	Not estimable^a^
Reduced appetite	1.11 (0.90, 1.37), 0.3465
Concentration difficulties	1.64 (1.34, 2.00), <0.0001
Lassitude	2.06 (1.70, 2.50), <0.0001
Inability to feel	1.84 (1.52, 2.21), <0.0001
Pessimistic thoughts	1.58 (1.31, 1.89), <0.0001
Suicidal thoughts	1.44 (1.05, 1.98), 0.0229

*Note*: Full analysis set in ESCAPE-TRD; MADRS item scores were evaluated as dichotomous outcomes for patients with a baseline item score of ≥2, “*n*” values are reported in Table 1.

a The Kaplan–Meier curves for reduced sleep crossed over; therefore, the hazard ratios were not proportional over time, violating the necessary assumption for the Cox proportional hazards model. Hence, hazard ratios for reduced sleep could not be estimated. CI, confidence interval; NS, nasal spray; XR, extended release.

Esketamine NS demonstrated the greatest benefit over quetiapine XR in resolving apparent sadness (HR: 1.94), reported sadness (HR: 1.96), lassitude (HR: 2.06), and inability to feel (HR: 1.84; Table 2 and Figure 5A–D [all *p* < 0.0001]). Similarly, esketamine NS also demonstrated a benefit in reducing inner tension, concentration difficulties, and pessimistic thoughts with HRs of 1.49, 1.64, and 1.58 (all *p* < 0.0001), respectively (Table 2 and Figure 6A–C). Likewise, esketamine NS treatment demonstrated a benefit over quetiapine XR in the resolution of suicidal thoughts (HR: 1.44; *p* < 0.05; Table 2 and Figure 6D).

**Figure 7.**
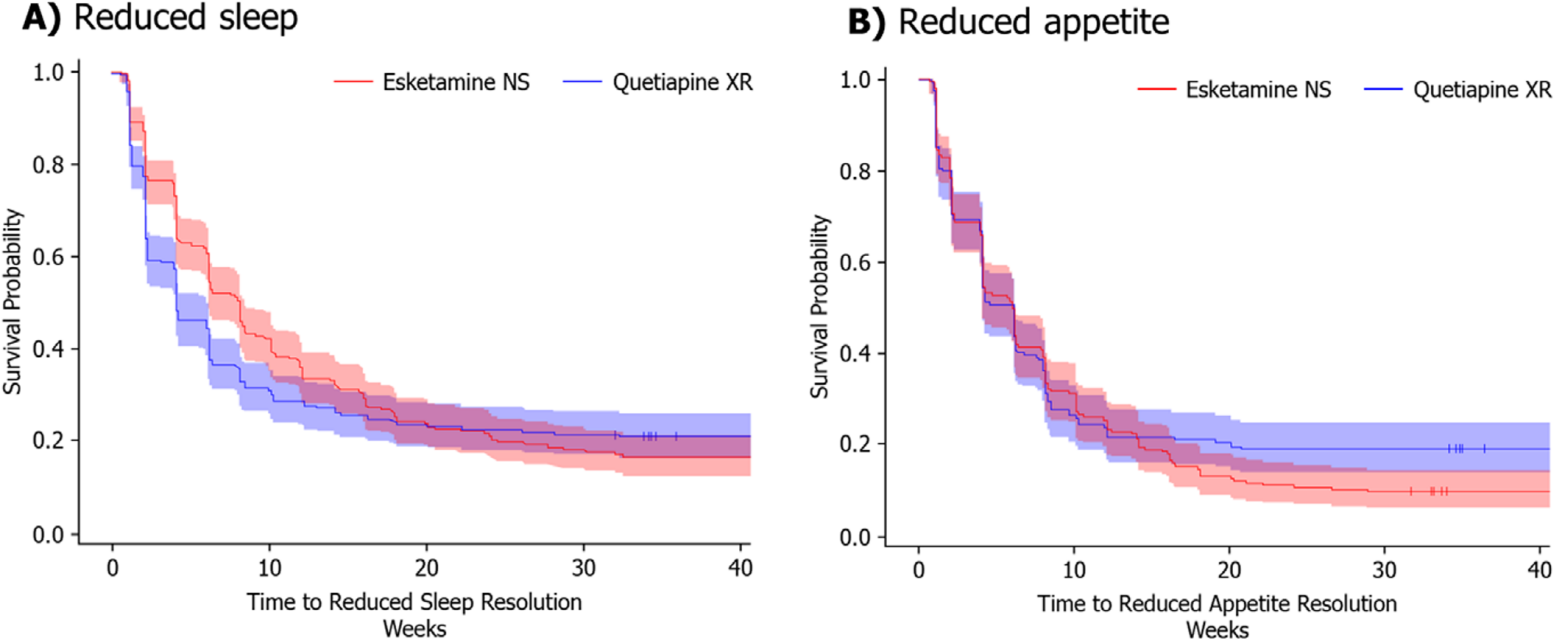
Time to symptom resolution for reduced sleep (A) and reduced appetite (B). Full analysis set in ESCAPE-TRD; MADRS item scores were evaluated as dichotomous outcomes for patients with a baseline item score of ≥2; “*n*” values are reported in Table 1. Survival probabilities represent the estimated probability that the symptom is not resolved over time across items in MADRS, with 95% confidence intervals; MADRS, Montgomery-Åsberg Depression Rating Scale; NS, nasal spray; XR, extended release. Figure 7. long description.

Similar rates of improvement in sleep were observed across treatment groups at any point up to Week 20 (Figure 7A). Though from Week 20 to 32, a slightly greater proportion of patients who received esketamine NS exhibited symptom resolution than in the quetiapine XR group (Figure 7A). As the Kaplan–Meier curves for reduced sleep crossed over during the maintenance phase, HRs for reduced sleep were not proportional over time, violating the necessary assumption for the Cox proportional hazards model (Figure 7A). Consequently, HRs for reduced sleep could not be estimated (Table 2).

Kaplan–Meier curves for appetite were similar across treatment arms, with esketamine NS demonstrating a slightly greater resolution of reduced appetite symptoms during the maintenance phase (Figure 7B). However, the overall difference between treatment groups in the resolution of appetite symptoms was not statistically significant (HR: 1.11; *p* = 0.3465; Table 2 and Figure 7B). The proportions of patients who achieved resolution of symptoms across MADRS items are reported to Week 32 (Supplementary Tables S1–S10).

## Discussion

Patients with TRD often experience poor treatment outcomes and may remain on treatments for long periods despite a lack of response [21, 22, 27]. The high prevalence of most symptoms at baseline in ESCAPE-TRD underlines the extensive burden of TRD, highlighting the need for treatments to address the full spectrum of symptoms. Indeed, this burden demonstrates the need for novel treatments that offer stable, long-term remission and emphasizes the importance of understanding the optimal time to persist with a treatment before making treatment continuation decisions [21, 28].

The ESCAPE-TRD primary analyses demonstrated the overall superior efficacy of esketamine NS versus quetiapine XR [26]. In these secondary analyses, patient flow between outcomes, represented by linking bands between stacked bar charts, demonstrated that patients who received esketamine NS generally progressed toward improved outcomes over 32 weeks, with most transitions representing improvements in patients’ outcomes. From Week 10, the majority of patients remained within response or remission, with few patients transitioning to less favorable outcomes. Even patients who had a minimal or partial response in the short term demonstrated improvements over time. Moreover, the proportion of patients with minimal or partial response decreased over 32 weeks, showing that few patients remained on esketamine NS without demonstrating improvements. However, the likelihood of achieving response or remission with esketamine NS treatment over 32 weeks was greater for patients who showed a response, including minimal response, partial response, or response, at Week 4 compared with Week 8.

Evaluating the degree of patients’ early improvement with esketamine NS treatment can serve as a useful tool to inform treatment continuation or modification decisions. In this study, Week 4 and Week 8 were considered meaningful short-term evaluation points, consistent with real-world clinical practice and real-world evidence studies, which commonly assess endpoints at Weeks 4 to 12 [29–31]. Moreover, evaluation at Week 4 aligns with the end of the induction phase for esketamine NS in the treatment of TRD, according to the Summary of Product Characteristics, which recommends that “evidence of therapeutic benefit should be evaluated at the end of induction phase to determine need for continued treatment” [24].

Over 32 weeks, the achievement of remission with esketamine NS increased steadily. Although the proportions of minimal responders, partial responders, and responders fluctuated slightly over time, biweekly analyses revealed a consistent trend toward clinical improvement, with a progressive transition from minimal response and partial response to response and remission. Patients treated with esketamine NS showed more pronounced improvements compared with those receiving quetiapine XR, and substantial proportions of patients treated with esketamine NS achieved stable remission. In general, patients who achieved response or remission at Week 4/8 demonstrated higher rates of stable remission over time compared with Week 4/8 partial or minimal responders (Figure 4B–E and Supplementary Figure S3A–D). These results demonstrate the potential of esketamine NS to not only help patients achieve remission but also to maintain it. Moreover, Week 4 responders demonstrated higher remission rates than Week 8 responders, indicating that earlier improvement was associated with better long-term outcomes.

Short-term improvements with esketamine NS were associated with long-term response and remission, as high proportions of Week 4/8 responders proceeded to achieve remission at any point from Week 4 to 32 and Week 8 to 32, respectively. Patients who achieved response or even partial response at Week 4 and/or Week 8 were more likely to reach remission at any point from Week 4 to 32 or Week 8 to 32, respectively, compared with Week 4/8 minimal responders. These findings suggest that early symptom changes, even if modest, may serve as useful prognostic indicators to guide clinical decisions.

Interestingly, although Week 4 minimal responders showed lower remission rates at Week 32 than Week 4 partial responders or responders, many of these patients did achieve response or remission at some point from Week 4 to 32. Furthermore, smaller proportions of patients treated with esketamine NS discontinued treatment over 32 weeks versus patients who received quetiapine XR. These results align with previous findings and support the idea that small threshold improvements are clinically relevant, and discontinuation of antidepressant therapy may be associated with higher relapse rates and worse quality of life [19, 32, 33]. These findings underscore the risk of prematurely discontinuing a potentially beneficial treatment.

Symptomatic improvement and/or the achievement of remission (MADRS total score ≤10) may not only correlate with quality of life improvements, but also serve as a prerequisite for functional improvements [11, 22, 34]. In this analysis, patients demonstrated faster time to symptom resolution with esketamine NS over 32 weeks, across most MADRS items, compared with quetiapine XR, demonstrating esketamine NS’ potential as a comprehensive treatment option. Notably, esketamine NS demonstrated an HR of 1.44 versus quetiapine XR for resolution of suicidal thoughts, indicating its potential effectiveness in addressing this critical aspect of TRD. These results demonstrate that esketamine NS may improve outcomes by addressing a broad range of TRD symptoms. However, as *p*-values were not adjusted for multiplicity, symptom-level analyses are exploratory, and any inferences should be treated with caution.

A factor structure of the MADRS has been proposed, grouping symptoms into three categories: affective and anhedonic, anxiety and vegetative, and hopelessness [35]. Identifying core and noncore TRD symptoms could aid understanding of esketamine NS’ impact on distinct symptom domains. Esketamine NS treatment demonstrated HRs of 1.84 versus quetiapine XR for resolution of inability to feel and 2.06 versus quetiapine XR for lassitude, indicating a 1.84- and 2.06-fold higher probability of symptom resolution for these anhedonia-related symptoms, respectively. HRs >1 indicate that patients treated with esketamine NS had a higher probability of resolution of these symptoms versus quetiapine XR. These results are clinically meaningful given the profound effects of such symptoms on patients’ quality of life and the limited effect of conventional antidepressants on anhedonia [36, 37]. These findings suggest that esketamine NS may be more effective in resolving “core” TRD symptoms, such as anhedonia and apparent sadness (or “depressed mood”), while quetiapine XR showed comparable efficacy in symptoms that may be considered as “non-core,” such as reduced sleep and appetite [11, 38]. Although quetiapine XR led to earlier improvement in sleep, esketamine NS showed similar resolution over the full 32-week period, suggesting that in the absence of early symptom resolution, continued esketamine NS treatment can still lead to meaningful clinical improvement in sleep. Moreover, it is important to consider whether early sleep improvements with quetiapine XR may be attributable to its sedative effects rather than its antidepressant properties [39, 40]. In contrast, although esketamine NS may cause sleepiness or sedation following administration, these adverse events are typically mild and transient; as such, the sedative effects of esketamine NS are likely to have little effect on patients’ sleep [26, 39, 41].

The lack of clinical consensus regarding the most effective treatment pathway for TRD underscores the need for well-tolerated, effective treatments to facilitate sustained remission and overall symptom alleviation. These findings may guide clinicians in tailoring treatments to support patients in achieving and maintaining remission. Further research to identify specific predictors of long-term outcomes may be beneficial [42].

### Strengths and limitations

A strength of these analyses is that symptom resolution data reported a broad range of symptoms; consistent results were observed, with esketamine NS providing symptom resolution across all MADRS items. A further strength is the illustration of individual patient trajectories and transitions between different outcomes over time with esketamine NS, demonstrating patients’ fluctuation between outcomes and enhancing the understanding gained from assessing overall proportions of patients achieving outcomes at each visit. Alluvial diagrams presented in this study enable focused analysis of patient trajectories. For instance, they facilitate the tracing of different subsets of patients across time points; for example, from observing the transitions of the 27.1% of patients who achieved remission at Week 8, remission was generally maintained for most patients at the next time point. Maintenance of symptom resolution at the item level was not assessed; however, overall outcome stability and achievement of stable remission with esketamine NS are reported, offering clinically relevant information on the long-term patient trajectories.

A notable limitation is the absence of adjustment for potential confounders, such as the use of benzodiazepines or the time spent with healthcare professionals. The lower baseline prevalence of suicidal thoughts compared with other symptoms is attributable to ESCAPE-TRD’s exclusion criteria. As a result, this patient subgroup was smaller, limiting statistical power for that domain. Future studies incorporating targeted subgroup analyses may elucidate the treatment effects on suicidal ideation more robustly.

## Conclusions

These secondary analyses demonstrate that most patients treated with esketamine NS achieved continuously improved outcomes over 32 weeks and provide evidence that short-term response is associated with more favorable and sustained long-term outcomes; this may be useful in guiding treatment continuation decisions. Improvements were observed even among Week 4 partial or minimal responders, reinforcing the value of ongoing esketamine NS treatment. Moreover, esketamine NS demonstrated a homogenous positive impact across the full spectrum of reported symptoms in patients with TRD.

## Supporting information

10.1192/j.eurpsy.2026.12229.sm001 Young et al. supplementary material Young et al. supplementary material

## Data Availability

The data sharing policy of Johnson & Johnson is available at https://innovativemedicine.jnj.com/our-innovation/clinical-trials/transparency. As noted on this site, requests for access to the study data can be submitted through the Yale Open Data Access (YODA) Project site at http://yoda.yale.edu.

## Supplementary material

The supplementary material for this article can be found at http://doi.org/10.1192/j.eurpsy.2026.12229.

## Long descriptions

### [Table 1] Long description

The table consists of four columns: Item, Esketamine N S plus S S R I or S N R I (N equals 336), Quetiapine X R plus S S R I or S N R I (N equals 339), and Overall (N equals 675).

Data is presented as a ratio and percentage for each symptom:

* Apparent sadness: Esketamine 324/336 (96.4 percent), Quetiapine 331/339 (97.6 percent), Overall 655/675 (97.0 percent).

* Reported sadness: Esketamine 333/336 (99.1 percent), Quetiapine 337/339 (99.4 percent), Overall 670/675 (99.3 percent).

* Inner tension: Esketamine 313/336 (93.2 percent), Quetiapine 321/339 (94.7 percent), Overall 634/675 (93.9 percent).

* Reduced sleep: Esketamine 289/336 (86.0 percent), Quetiapine 297/339 (87.6 percent), Overall 586/675 (86.8 percent).

* Reduced appetite: Esketamine 205/336 (61.0 percent), Quetiapine 199/339 (58.7 percent), Overall 404/675 (59.9 percent).

* Concentration difficulties: Esketamine 329/336 (97.9 percent), Quetiapine 322/339 (95.0 percent), Overall 651/675 (96.4 percent).

* Lassitude: Esketamine 329/336 (97.9 percent), Quetiapine 322/339 (95.0 percent), Overall 651/675 (96.4 percent).

* Inability to feel: Esketamine 325/336 (96.7 percent), Quetiapine 323/339 (95.3 percent), Overall 648/675 (96.0 percent).

* Pessimistic thoughts: Esketamine 306/336 (91.1 percent), Quetiapine 298/339 (87.9 percent), Overall 604/675 (89.5 percent).

* Suicidal thoughts: Esketamine 94/336 (28.0 percent), Quetiapine 93/339 (27.4 percent), Overall 187/675 (27.7 percent).

Navigate back to [Table 1].

### [Figure 1] Long description

The x-axis represents time from Baseline to Week 32. The y-axis represents the number of patients (n equals 336).

Vertical bars at each time point (Weeks 1, 2, 4, 6, 8, 10, 12, 14, 16, 18, 20, 22, 24, 26, 28, 30, and 32) are divided into five color-coded categories:

* Dark Purple (Top): Remission. Starts at 8 patients in Week 1 and increases steadily to 167 patients by Week 32.

* Teal: Response. Fluctuates from 11 patients in Week 1, peaking at 118 in Week 4, and settling at 55 by Week 32.

* Light Green: Partial Response. Starts at 73 in Week 1, peaking at 127 in Week 2, and decreasing to 26 by Week 32.

* Orange: Minimal Response. Starts at 240 at Week 1 and decreases significantly to 10 by Week 32.

* Light Pink (Bottom): Off study treatment. Starts at 4 in Week 1 and grows to 78 by Week 32.

Flowing bands between the bars illustrate how individual patients move between these states. The most prominent trend is the widening of the dark purple Remission band and the light pink Off study treatment band over time, while the orange Minimal Response band narrows sharply after the first two weeks.

Navigate back to [Figure 1].

### [Figure 2] Long description

A central donut chart titled Week 4 Outcomes is divided into five segments. Starting from the top and moving clockwise:

* Off study treatment: 5.7 percent, n equals 19 (light purple).

* Minimal response: 23.5 percent, n equals 79 (orange).

* Partial response: 35.1 percent, n equals 118 (light green).

* Response: 22.9 percent, n equals 77 (teal).

* Remission: 12.8 percent, n equals 43 (dark purple).

Three bar charts are linked to specific segments of the donut chart:

1. Top-Left (linked to Response): A single dark purple bar shows that for patients with a Response at Week 4, 89.6 percent (69 out of 77) achieved Remission at any point from Week 4 to 32.

2. Top-Right (linked to Minimal response): Two bars show outcomes for patients with Minimal response at Week 4. The teal bar indicates 69.6 percent (55 out of 79) achieved Response at any point from Week 4 to 32. The dark purple bar indicates 49.4 percent (39 out of 79) achieved Remission in the same period.

3. Bottom-Right (linked to Partial response): Two bars show outcomes for patients with Partial response at Week 4. The teal bar indicates 88.1 percent (104 out of 118) achieved Response at any point from Week 4 to 32. The dark purple bar indicates 66.1 percent (78 out of 118) achieved Remission in the same period.

All bar charts use a Y-axis representing Proportion of patients in percent from 0 to 100.

Navigate back to [Figure 2].

### [Figure 3] Long description

A central donut chart titled Week 8 Outcomes is divided into five segments. Moving clockwise from the top:

* Off study treatment (light pink): 10.1 percent, n equals 34.

* Minimal response (orange): 13.4 percent, n equals 45.

* Partial response (light green): 24.7 percent, n equals 83.

* Response (teal): 24.7 percent, n equals 83.

* Remission (dark purple): 27.1 percent, n equals 91.

Three bar charts are connected to specific segments of the donut chart to show outcomes from Week 8 to 32:

1. Top-left chart (connected to Response): A single dark purple bar shows that for patients with a Response at Week 8, 78.3 percent (65 out of 83) achieved Remission at any point from Week 8 to 32.

2. Top-right chart (connected to Minimal response): Two bars show that for patients with a Minimal response at Week 8, 51.1 percent (23 out of 45) achieved Response (teal bar) and 33.3 percent (15 out of 45) achieved Remission (dark purple bar) at any point from Week 8 to 32.

3. Bottom-right chart (connected to Partial response): Two bars show that for patients with a Partial response at Week 8, 88.0 percent (73 out of 83) achieved Response (teal bar) and 63.9 percent (53 out of 83) achieved Remission (dark purple bar) at any point from Week 8 to 32.

All bar charts use a Y-axis representing Proportion of patients in percent from 0 to 100.

Navigate back to [Figure 3].

### [Figure 4] Long description

A multi-panel figure containing five Sankey diagrams labeled A through E. All panels share a common legend at the bottom: Remission (dark purple), Remission stable (dark purple with red outline), Response (teal), Partial Response (green), Minimal Response (orange), and Off study treatment (light purple).

Panel A: Outcome stability from baseline. Starting with 336 patients at baseline, the flow shows a gradual increase in the Remission stable category (red outlined) reaching 167 patients by Week 32. The Off study treatment group grows steadily to 78 patients. Other categories fluctuate as patients transition between response levels.

Panel B: Outcome stability following remission at Week 4. Focuses on a subset of 43 patients in remission at Week 4. By Week 32, 33 of these patients are in stable remission, while others have transitioned to response, partial response, or off treatment.

Panel C: Outcome stability following remission at Week 8. Tracks 91 patients in remission at Week 8. The stable remission group grows to 65 patients by Week 32, with a significant portion of the remainder moving to the off study treatment category (11 patients).

Panel D: Outcome stability following response at Week 4. Tracks 77 patients who achieved response at Week 4. By Week 32, 54 are in stable remission, while others are distributed across response (7), partial response (2), and off treatment (13).

Panel E: Outcome stability following response at Week 8. Tracks 83 patients who achieved response at Week 8. By Week 32, 55 are in stable remission, 19 are in response, 4 are in partial response, and 5 are off treatment.

Across all panels, the red-outlined ‘Remission (stable)’ blocks indicate that once a patient enters this state, they remain in remission through Week 32 according to L O C F methodology.

Navigate back to [Figure 4].

### [Figure 5] Long description

A four-panel set of Kaplan-Meier survival curves. Each graph shares a common Y-axis labeled Survival Probability from 0.0 to 1.0 and an X-axis labeled Weeks from 0 to 40. In all panels, Esketamine N S is represented by a red line and Quetiapine X R by a blue line, both with shaded 95 percent confidence intervals.

* Panel A, Apparent Sadness: The red line drops more steeply than the blue line, reaching a survival probability of approximately 0.2 by week 32, while the blue line remains higher at approximately 0.45.

* Panel B, Reported Sadness: Similar to panel A, the red line shows a faster resolution of symptoms, plateauing near 0.25 after week 30, whereas the blue line plateaus near 0.5.

* Panel C, Lassitude: The red line descends steadily to approximately 0.2 by week 30. The blue line follows a higher trajectory, ending near 0.45 at week 40.

* Panel D, Inability to feel: The red line shows a rapid decline in the first 15 weeks, stabilising around 0.2. The blue line shows a more gradual decline, ending at approximately 0.4.

In all four symptoms, the Esketamine N S group shows a lower survival probability over time compared to the Quetiapine X R group, indicating a faster time to symptom resolution.

Navigate back to [Figure 5].

### [Table 2] Long description

The table consists of two columns: Item and Hazard ratio (95% C I), p-value.

* Apparent sadness: 1.94 (1.61, 2.35), p < .0001.

* Reported sadness: 1.96 (1.62, 2.38), p < .0001.

* Inner tension: 1.49 (1.24, 1.79), p < .0001.

* Reduced sleep: Not estimable due to crossing Kaplan-Meier curves.

* Reduced appetite: 1.11 (0.90, 1.37), p = 0.3465.

* Concentration difficulties: 1.64 (1.34, 2.00), p < .0001.

* Lassitude: 2.06 (1.70, 2.50), p < .0001.

* Inability to feel: 1.84 (1.52, 2.21), p < .0001.

* Pessimistic thoughts: 1.58 (1.31, 1.89), p < .0001.

* Suicidal thoughts: 1.44 (1.05, 1.98), p = 0.0229.

Navigate back to [Table 2].

### [Figure 6] Long description

A four-panel set of line graphs. Each graph shares a Y-axis labeled Survival Probability from 0.0 to 1.0 and an X-axis labeled Time to [Symptom] Resolution in Weeks from 0 to 40. All panels show two stepped lines with shaded 95 percent confidence intervals: a red line for Esketamine N S and a blue line for Quetiapine X R.

* Panel A, Inner tension: Both lines start at 1.0. The red line drops more steeply, reaching a probability of approximately 0.2 by week 40. The blue line remains higher, ending near 0.35.

* Panel B, Concentration difficulties: The red line shows a steady decline to approximately 0.3. The blue line follows a similar but higher trajectory, ending near 0.5.

* Panel C, Pessimistic thoughts: The red line drops sharply in the first 10 weeks and plateaus near 0.15. The blue line stays consistently above it, ending near 0.3.

* Panel D, Suicidal thoughts: Both lines show the most rapid decline of all panels within the first 5 weeks. The red line reaches a low probability of approximately 0.1, while the blue line levels off near 0.2.

In all four panels, the Esketamine N S group (red) consistently shows a lower survival probability than the Quetiapine X R group (blue), indicating a faster time to symptom resolution.

Navigate back to [Figure 6].

### [Figure 7] Long description

Two line graphs arranged horizontally. Both share a y-axis labeled Survival Probability from 0.0 to 1.0 and an x-axis labeled Weeks from 0 to 40.

Panel A, titled Reduced sleep, shows the time to resolution for sleep symptoms. The x-axis is Time to Reduced Sleep Resolution Weeks. The Esketamine N S group, represented by a red line with a light red shaded confidence interval, shows a slower initial decline in survival probability compared to the Quetiapine X R group, represented by a blue line with a light blue shaded confidence interval. By week 10, the probability for Esketamine N S is approximately 0.4, while Quetiapine X R is approximately 0.3. The lines converge and plateau near 0.2 by week 30.

Panel B, titled Reduced appetite, shows the time to resolution for appetite symptoms. The x-axis is Time to Reduced Appetite Resolution Weeks. Both the red Esketamine N S line and the blue Quetiapine X R line follow a very similar rapid downward trajectory until week 10, reaching a probability of approximately 0.3. After week 15, the Esketamine N S line drops slightly lower than the Quetiapine X R line, with both plateauing between 0.1 and 0.2 through week 40. Small vertical tick marks on the lines indicate censored data points occurring after week 30.

Navigate back to [Figure 7].
