# Supplementary material for: Trajectory of response to esketamine nasal spray for treatment resistant depression: Findings from ESCAPE-TRD
Source: Eur Psychiatry. 2026 Jun 16;69(1):e74. doi: 10.1192/j.eurpsy.2026.12229 (PMC13420147; doi:10.1192/j.eurpsy.2026.12229)
Supplement: Young et al. supplementary material [file S0924933826122299sup001.docx]

SUPPLEMENTARY APPENDIX

APPENDIX 1. Supplementary Methods

For proportions of patients with resolution of symptoms based on MADRS, reported in Supplementary Tables 1–10, treatment discontinuations were imputed as negative outcomes (i.e. symptom not resolved; non-responder imputation [NRI]). Missing data for patients still on treatment imputed using LOCF. Odds ratio and p-values from Cochran–Mantel–Haenszel chi-square tests were stratified by age (18–≤64 years; 65–≤74 years) and number of prior treatment failures in the current major depressive episode (2 or ≥3).

SUPPLEMENTARY MATERIALS

Supplementary Table 1. Proportion of patients with resolution of apparent sadness based on MADRS (NRI)

| **Week** | **Esketamine NS + SSRI/SNRI (N=336)** | **Quetiapine XR + SSRI/SNRI (N=340)** | **Odds Ratio** | **p-value** |
| --- | --- | --- | --- | --- |
| 1 | 27 (8.04%) | 10 (2.94%) | 2.926 [1.392; 6.150] | 0.0033 |
| 2 | 57 (16.96%) | 24 (7.06%) | 2.658 [1.615; 4.377] | <0.0001 |
| 4 | 93 (27.68%) | 49 (14.41%) | 2.270 [1.547; 3.331] | <0.0001 |
| 6 | 118 (35.12%) | 54 (15.88%) | 2.872 [1.992; 4.141] | <0.0001 |
| 8 | 122 (36.31%) | 71 (20.88%) | 2.167 [1.537; 3.056] | <0.0001 |
| 10 | 148 (44.05%) | 80 (23.53%) | 2.582 [1.852; 3.598] | <0.0001 |
| 12 | 155 (46.13%) | 80 (23.53%) | 2.808 [2.016; 3.909] | <0.0001 |
| 14 | 163 (48.51%) | 86 (25.29%) | 2.858 [2.057; 3.971] | <0.0001 |
| 16 | 161 (47.92%) | 99 (29.12%) | 2.275 [1.652; 3.133] | <0.0001 |
| 18 | 162 (48.21%) | 94 (27.65%) | 2.468 [1.790; 3.404] | <0.0001 |
| 20 | 163 (48.51%) | 103 (30.29%) | 2.213 [1.609; 3.043] | <0.0001 |
| 22 | 167 (49.70%) | 105 (30.88%) | 2.263 [1.647; 3.108] | <0.0001 |
| 24 | 169 (50.30%) | 104 (30.59%) | 2.317 [1.690; 3.177] | <0.0001 |
| 26 | 163 (48.51%) | 112 (32.94%) | 1.948 [1.423; 2.666] | <0.0001 |
| 28 | 169 (50.30%) | 119 (35.00%) | 1.891 [1.387; 2.577] | <0.0001 |
| 30 | 185 (55.06%) | 121 (35.59%) | 2.230 [1.636; 3.039] | <0.0001 |
| 32 | 191 (56.85%) | 120 (35.29%) | 2.434 [1.784; 3.322] | <0.0001 |

Full methodology reported in **Appendix 1**. MADRS: Montgomery-Åsberg Depression Rating Scale; NRI: non-responder imputation; NS: nasal spray; SNRI: serotonin norepinephrine reuptake inhibitor; SSRI: selective serotonin reuptake inhibitor; XR: extended release.

| **Week** | **Esketamine NS + SSRI/SNRI (N=336)** | **Quetiapine XR + SSRI/SNRI (N=340)** | **Odds Ratio** | **p-value** |
| --- | --- | --- | --- | --- |
| 1 | 16 (4.76%) | 5 (1.47%) | 3.373 [1.217; 9.348] | 0.0137 |
| 2 | 33 (9.82%) | 21 (6.18%) | 1.648 [0.933; 2.912] | 0.0834 |
| 4 | 76 (22.62%) | 42 (12.35%) | 2.080 [1.378; 3.141] | 0.0004 |
| 6 | 103 (30.65%) | 56 (16.47%) | 2.258 [1.560; 3.269] | <0.0001 |
| 8 | 116 (34.52%) | 70 (20.59%) | 2.048 [1.447; 2.898] | <0.0001 |
| 10 | 143 (42.56%) | 82 (24.12%) | 2.364 [1.696; 3.295] | <0.0001 |
| 12 | 146 (43.45%) | 74 (21.76%) | 2.794 [1.993; 3.916] | <0.0001 |
| 14 | 143 (42.56%) | 77 (22.65%) | 2.591 [1.849; 3.631] | <0.0001 |
| 16 | 141 (41.96%) | 90 (26.47%) | 2.030 [1.466; 2.813] | <0.0001 |
| 18 | 139 (41.37%) | 90 (26.47%) | 1.972 [1.424; 2.730] | <0.0001 |
| 20 | 148 (44.05%) | 97 (28.53%) | 2.010 [1.456; 2.775] | <0.0001 |
| 22 | 146 (43.45%) | 105 (30.88%) | 1.743 [1.269; 2.395] | 0.0006 |
| 24 | 150 (44.64%) | 95 (27.94%) | 2.113 [1.531; 2.916] | <0.0001 |
| 26 | 155 (46.13%) | 107 (31.47%) | 1.899 [1.384; 2.606] | <0.0001 |
| 28 | 161 (47.92%) | 114 (33.53%) | 1.841 [1.348; 2.515] | 0.0001 |
| 30 | 163 (48.51%) | 105 (30.88%) | 2.117 [1.545; 2.899] | <0.0001 |
| 32 | 170 (50.60%) | 109 (32.06%) | 2.191 [1.601; 2.997] | <0.0001 |

Supplementary Table 2. Proportion of patients with resolution of reported sadness based on MADRS (NRI)

Full methodology reported in **Appendix 1**. MADRS: Montgomery-Åsberg Depression Rating Scale; NRI: non-responder imputation; NS: nasal spray; SNRI: serotonin norepinephrine reuptake inhibitor; SSRI: selective serotonin reuptake inhibitor; XR: extended release.

Supplementary Table 3. Proportion of patients with resolution of inner tension based on MADRS (NRI)

| **Week** | **Esketamine NS + SSRI/SNRI (N=336)** | **Quetiapine XR + SSRI/SNRI (N=340)** | **Odds Ratio** | **p-value** |
| --- | --- | --- | --- | --- |
| 1 | 44 (13.10%) | 29 (8.53%) | 1.607 [0.978; 2.640] | 0.0602 |
| 2 | 61 (18.15%) | 46 (13.53%) | 1.418 [0.934; 2.154] | 0.1007 |
| 4 | 86 (25.60%) | 59 (17.35%) | 1.654 [1.137; 2.406] | 0.0082 |
| 6 | 116 (34.52%) | 78 (22.94%) | 1.794 [1.274; 2.526] | 0.0008 |
| 8 | 126 (37.50%) | 101 (29.71%) | 1.431 [1.036; 1.977] | 0.0294 |
| 10 | 136 (40.48%) | 105 (30.88%) | 1.534 [1.116; 2.109] | 0.0084 |
| 12 | 136 (40.48%) | 100 (29.41%) | 1.658 [1.201; 2.289] | 0.0020 |
| 14 | 139 (41.37%) | 96 (28.24%) | 1.815 [1.315; 2.506] | 0.0003 |
| 16 | 134 (39.88%) | 102 (30.00%) | 1.569 [1.138; 2.163] | 0.0058 |
| 18 | 144 (42.86%) | 105 (30.88%) | 1.714 [1.245; 2.360] | 0.0009 |
| 20 | 145 (43.15%) | 108 (31.76%) | 1.674 [1.216; 2.305] | 0.0016 |
| 22 | 147 (43.75%) | 113 (33.24%) | 1.614 [1.173; 2.221] | 0.0033 |
| 24 | 150 (44.64%) | 123 (36.18%) | 1.460 [1.065; 2.001] | 0.0186 |
| 26 | 158 (47.02%) | 128 (37.65%) | 1.494 [1.096; 2.037] | 0.0112 |
| 28 | 150 (44.64%) | 126 (37.06%) | 1.391 [1.019; 1.898] | 0.0381 |
| 30 | 152 (45.24%) | 125 (36.76%) | 1.441 [1.056; 1.966] | 0.0212 |
| 32 | 147 (43.75%) | 118 (34.71%) | 1.485 [1.085; 2.031] | 0.0135 |

Full methodology reported in **Appendix 1**. MADRS: Montgomery-Åsberg Depression Rating Scale; NRI: non-responder imputation; NS: nasal spray; SNRI: serotonin norepinephrine reuptake inhibitor; SSRI: selective serotonin reuptake inhibitor; XR: extended release.

| **Week** | **Esketamine NS + SSRI/SNRI (N=336)** | **Quetiapine XR + SSRI/SNRI (N=340)** | **Odds Ratio** | **p-value** |
| --- | --- | --- | --- | --- |
| 1 | 65 (19.35%) | 87 (25.59%) | 0.698 [0.485; 1.004] | 0.0517 |
| 2 | 93 (27.68%) | 136 (40.00%) | 0.577 [0.418; 0.796] | 0.0008 |
| 4 | 114 (33.93%) | 146 (42.94%) | 0.682 [0.500; 0.931] | 0.0158 |
| 6 | 120 (35.71%) | 163 (47.94%) | 0.605 [0.445; 0.823] | 0.0013 |
| 8 | 136 (40.48%) | 172 (50.59%) | 0.665 [0.491; 0.902] | 0.0086 |
| 10 | 133 (39.58%) | 166 (48.82%) | 0.689 [0.507; 0.934] | 0.0165 |
| 12 | 137 (40.77%) | 160 (47.06%) | 0.777 [0.573; 1.054] | 0.1058 |
| 14 | 142 (42.26%) | 162 (47.65%) | 0.808 [0.596; 1.096] | 0.1707 |
| 16 | 161 (47.92%) | 159 (46.76%) | 1.054 [0.779; 1.428] | 0.7329 |
| 18 | 165 (49.11%) | 160 (47.06%) | 1.092 [0.807; 1.478] | 0.5702 |
| 20 | 154 (45.83%) | 159 (46.76%) | 0.968 [0.715; 1.312] | 0.8366 |
| 22 | 153 (45.54%) | 156 (45.88%) | 0.993 [0.733; 1.346] | 0.9647 |
| 24 | 155 (46.13%) | 161 (47.35%) | 0.957 [0.707; 1.296] | 0.7780 |
| 26 | 155 (46.13%) | 160 (47.06%) | 0.969 [0.715; 1.312] | 0.8372 |
| 28 | 164 (48.81%) | 160 (47.06%) | 1.077 [0.797; 1.457] | 0.6289 |
| 30 | 157 (46.73%) | 150 (44.12%) | 1.115 [0.823; 1.510] | 0.4836 |
| 32 | 159 (47.32%) | 151 (44.41%) | 1.131 [0.834; 1.533] | 0.4291 |

Supplementary Table 4. Proportion of patients with resolution of reduced sleep based on MADRS (NRI)

Full methodology reported in **Appendix 1**. MADRS: Montgomery-Åsberg Depression Rating Scale; NRI: non-responder imputation; NS: nasal spray; SNRI: serotonin norepinephrine reuptake inhibitor; SSRI: selective serotonin reuptake inhibitor; XR: extended release.

| **Week** | **Esketamine NS + SSRI/SNRI (N=336)** | **Quetiapine XR + SSRI/SNRI (N=340)** | **Odds Ratio** | **p-value** |
| --- | --- | --- | --- | --- |
| 1 | 152 (45.24%) | 162 (47.65%) | 0.906 [0.669; 1.225] | 0.5214 |
| 2 | 169 (50.30%) | 166 (48.82%) | 1.059 [0.784; 1.431] | 0.7092 |
| 4 | 199 (59.23%) | 186 (54.71%) | 1.204 [0.888; 1.632] | 0.2321 |
| 6 | 214 (63.69%) | 200 (58.82%) | 1.230 [0.902; 1.677] | 0.1908 |
| 8 | 216 (64.29%) | 203 (59.71%) | 1.220 [0.893; 1.666] | 0.2118 |
| 10 | 226 (67.26%) | 198 (58.24%) | 1.483 [1.083; 2.031] | 0.0142 |
| 12 | 223 (66.37%) | 201 (59.12%) | 1.368 [1.001; 1.870] | 0.0493 |
| 14 | 233 (69.35%) | 195 (57.35%) | 1.700 [1.237; 2.335] | 0.0010 |
| 16 | 241 (71.73%) | 197 (57.94%) | 1.864 [1.351; 2.573] | 0.0001 |
| 18 | 234 (69.64%) | 191 (56.18%) | 1.822 [1.324; 2.507] | 0.0002 |
| 20 | 238 (70.83%) | 194 (57.06%) | 1.849 [1.342; 2.545] | 0.0002 |
| 22 | 235 (69.94%) | 198 (58.24%) | 1.686 [1.225; 2.320] | 0.0013 |
| 24 | 226 (67.26%) | 195 (57.35%) | 1.549 [1.130; 2.125] | 0.0066 |
| 26 | 228 (67.86%) | 187 (55.00%) | 1.757 [1.281; 2.410] | 0.0005 |
| 28 | 231 (68.75%) | 184 (54.12%) | 1.893 [1.379; 2.597] | <0.0001 |
| 30 | 226 (67.26%) | 182 (53.53%) | 1.804 [1.319; 2.469] | 0.0002 |
| 32 | 221 (65.77%) | 181 (53.24%) | 1.707 [1.250; 2.331] | 0.0008 |

Supplementary Table 5. Proportion of patients with resolution of reduced appetite based on MADRS (NRI)

Full methodology reported in **Appendix 1**. MADRS: Montgomery-Åsberg Depression Rating Scale; NRI: non-responder imputation; NS: nasal spray; SNRI: serotonin norepinephrine reuptake inhibitor; SSRI: selective serotonin reuptake inhibitor; XR: extended release.

| **Week** | **Esketamine NS + SSRI/SNRI (N=336)** | **Quetiapine XR + SSRI/SNRI (N=340)** | **Odds Ratio** | **p-value** |
| --- | --- | --- | --- | --- |
| 1 | 18 (5.36%) | 14 (4.12%) | 1.313 [0.644; 2.679] | 0.4511 |
| 2 | 36 (10.71%) | 27 (7.94%) | 1.375 [0.814; 2.322] | 0.2321 |
| 4 | 51 (15.18%) | 39 (11.47%) | 1.370 [0.879; 2.136] | 0.1610 |
| 6 | 67 (19.94%) | 47 (13.82%) | 1.542 [1.028; 2.314] | 0.0353 |
| 8 | 92 (27.38%) | 58 (17.06%) | 1.814 [1.256; 2.621] | 0.0014 |
| 10 | 109 (32.44%) | 66 (19.41%) | 1.988 [1.399; 2.824] | 0.0001 |
| 12 | 110 (32.74%) | 70 (20.59%) | 1.878 [1.326; 2.659] | 0.0003 |
| 14 | 118 (35.12%) | 74 (21.76%) | 1.941 [1.381; 2.727] | 0.0001 |
| 16 | 116 (34.52%) | 73 (21.47%) | 1.937 [1.375; 2.730] | 0.0001 |
| 18 | 132 (39.29%) | 68 (20.00%) | 2.623 [1.856; 3.708] | <0.0001 |
| 20 | 124 (36.90%) | 81 (23.82%) | 1.887 [1.350; 2.638] | 0.0002 |
| 22 | 126 (37.50%) | 81 (23.82%) | 1.928 [1.381; 2.692] | 0.0001 |
| 24 | 122 (36.31%) | 90 (26.47%) | 1.583 [1.141; 2.196] | 0.0057 |
| 26 | 130 (38.69%) | 81 (23.82%) | 2.017 [1.447; 2.811] | <0.0001 |
| 28 | 132 (39.29%) | 97 (28.53%) | 1.637 [1.185; 2.261] | 0.0027 |
| 30 | 135 (40.18%) | 96 (28.24%) | 1.709 [1.239; 2.356] | 0.0010 |
| 32 | 127 (37.80%) | 101 (29.71%) | 1.446 [1.049; 1.994] | 0.0246 |

Supplementary Table 6. Proportion of patients with resolution of concentration difficulties based on MADRS (NRI)

Full methodology reported in **Appendix 1**. MADRS: Montgomery-Åsberg Depression Rating Scale; NRI: non-responder imputation; NS: nasal spray; SNRI: serotonin norepinephrine reuptake inhibitor; SSRI: selective serotonin reuptake inhibitor; XR: extended release.

| **Week** | **Esketamine NS + SSRI/SNRI (N=336)** | **Quetiapine XR + SSRI/SNRI (N=340)** | **Odds Ratio** | **p-value** |
| --- | --- | --- | --- | --- |
| 1 | 21 (6.25%) | 23 (6.76%) | 0.924 [0.501; 1.705] | 0.8019 |
| 2 | 42 (12.50%) | 27 (7.94%) | 1.667 [1.001; 2.777] | 0.0481 |
| 4 | 85 (25.30%) | 46 (13.53%) | 2.181 [1.465; 3.245] | 0.0001 |
| 6 | 98 (29.17%) | 62 (18.24%) | 1.854 [1.290; 2.664] | 0.0008 |
| 8 | 108 (32.14%) | 72 (21.18%) | 1.769 [1.250; 2.503] | 0.0012 |
| 10 | 127 (37.80%) | 69 (20.29%) | 2.380 [1.688; 3.355] | <0.0001 |
| 12 | 131 (38.99%) | 80 (23.53%) | 2.103 [1.505; 2.939] | <0.0001 |
| 14 | 119 (35.42%) | 77 (22.65%) | 1.913 [1.359; 2.692] | 0.0002 |
| 16 | 134 (39.88%) | 92 (27.06%) | 1.805 [1.304; 2.499] | 0.0004 |
| 18 | 136 (40.48%) | 79 (23.24%) | 2.295 [1.640; 3.210] | <0.0001 |
| 20 | 143 (42.56%) | 87 (25.59%) | 2.187 [1.576; 3.035] | <0.0001 |
| 22 | 150 (44.64%) | 91 (26.76%) | 2.233 [1.615; 3.088] | <0.0001 |
| 24 | 149 (44.35%) | 99 (29.12%) | 1.958 [1.424; 2.694] | <0.0001 |
| 26 | 145 (43.15%) | 97 (28.53%) | 1.941 [1.406; 2.679] | <0.0001 |
| 28 | 147 (43.75%) | 100 (29.41%) | 1.899 [1.379; 2.617] | <0.0001 |
| 30 | 158 (47.02%) | 102 (30.00%) | 2.094 [1.525; 2.876] | <0.0001 |
| 32 | 164 (48.81%) | 100 (29.41%) | 2.325 [1.691; 3.198] | <0.0001 |

Supplementary Table 7. Proportion of patients with resolution of lassitude based on MADRS (NRI)

Full methodology reported in **Appendix 1**. MADRS: Montgomery-Åsberg Depression Rating Scale; NRI: non-responder imputation; NS: nasal spray; SNRI: serotonin norepinephrine reuptake inhibitor; SSRI: selective serotonin reuptake inhibitor; XR: extended release.

Supplementary Table 8. Proportion of patients with resolution of inability to feel based on MADRS (NRI)

| **Week** | **Esketamine NS + SSRI/SNRI (N=336)** | **Quetiapine XR + SSRI/SNRI (N=340)** | **Odds Ratio** | **p-value** |
| --- | --- | --- | --- | --- |
| 1 | 28 (8.33%) | 16 (4.71%) | 1.840 [0.976; 3.471] | 0.0570 |
| 2 | 50 (14.88%) | 23 (6.76%) | 2.406 [1.432; 4.043] | 0.0007 |
| 4 | 79 (23.51%) | 46 (13.53%) | 1.964 [1.317; 2.929] | 0.0008 |
| 6 | 115 (34.23%) | 66 (19.41%) | 2.170 [1.527; 3.082] | <0.0001 |
| 8 | 140 (41.67%) | 71 (20.88%) | 2.727 [1.939; 3.835] | <0.0001 |
| 10 | 155 (46.13%) | 79 (23.24%) | 2.839 [2.038; 3.953] | <0.0001 |
| 12 | 159 (47.32%) | 93 (27.35%) | 2.406 [1.744; 3.319] | <0.0001 |
| 14 | 160 (47.62%) | 92 (27.06%) | 2.479 [1.796; 3.422] | <0.0001 |
| 16 | 167 (49.70%) | 96 (28.24%) | 2.545 [1.847; 3.506] | <0.0001 |
| 18 | 165 (49.11%) | 101 (29.71%) | 2.350 [1.706; 3.238] | <0.0001 |
| 20 | 164 (48.81%) | 103 (30.29%) | 2.238 [1.628; 3.077] | <0.0001 |
| 22 | 172 (51.19%) | 110 (32.35%) | 2.234 [1.631; 3.061] | <0.0001 |
| 24 | 174 (51.79%) | 126 (37.06%) | 1.848 [1.357; 2.516] | <0.0001 |
| 26 | 176 (52.38%) | 121 (35.59%) | 2.035 [1.489; 2.779] | <0.0001 |
| 28 | 175 (52.08%) | 125 (36.76%) | 1.883 [1.384; 2.563] | <0.0001 |
| 30 | 176 (52.38%) | 118 (34.71%) | 2.079 [1.525; 2.833] | <0.0001 |
| 32 | 186 (55.36%) | 122 (35.88%) | 2.241 [1.644; 3.056] | <0.0001 |

Full methodology reported in **Appendix 1**. MADRS: Montgomery-Åsberg Depression Rating Scale; NRI: non-responder imputation; NS: nasal spray; SNRI: serotonin norepinephrine reuptake inhibitor; SSRI: selective serotonin reuptake inhibitor; XR: extended release.

| **Week** | **Esketamine NS + SSRI/SNRI (N=336)** | **Quetiapine XR + SSRI/SNRI (N=340)** | **Odds Ratio** | **p-value** |
| --- | --- | --- | --- | --- |
| 1 | 65 (19.35%) | 64 (18.82%) | 1.038 [0.704; 1.530] | 0.8507 |
| 2 | 109 (32.44%) | 84 (24.71%) | 1.480 [1.055; 2.077] | 0.0233 |
| 4 | 147 (43.75%) | 105 (30.88%) | 1.761 [1.283; 2.419] | 0.0004 |
| 6 | 171 (50.89%) | 123 (36.18%) | 1.848 [1.355; 2.520] | <0.0001 |
| 8 | 165 (49.11%) | 135 (39.71%) | 1.474 [1.085; 2.003] | 0.0131 |
| 10 | 182 (54.17%) | 142 (41.76%) | 1.666 [1.227; 2.261] | 0.0010 |
| 12 | 185 (55.06%) | 138 (40.59%) | 1.823 [1.340; 2.481] | 0.0001 |
| 14 | 181 (53.87%) | 136 (40.00%) | 1.780 [1.308; 2.422] | 0.0002 |
| 16 | 192 (57.14%) | 137 (40.29%) | 2.021 [1.483; 2.756] | <0.0001 |
| 18 | 189 (56.25%) | 140 (41.18%) | 1.859 [1.368; 2.527] | <0.0001 |
| 20 | 189 (56.25%) | 144 (42.35%) | 1.772 [1.304; 2.409] | 0.0003 |
| 22 | 193 (57.44%) | 148 (43.53%) | 1.778 [1.308; 2.416] | 0.0002 |
| 24 | 198 (58.93%) | 148 (43.53%) | 1.902 [1.397; 2.591] | <0.0001 |
| 26 | 191 (56.85%) | 143 (42.06%) | 1.847 [1.357; 2.514] | <0.0001 |
| 28 | 194 (57.74%) | 141 (41.47%) | 1.947 [1.432; 2.647] | <0.0001 |
| 30 | 197 (58.63%) | 137 (40.29%) | 2.116 [1.555; 2.879] | <0.0001 |
| 32 | 203 (60.42%) | 137 (40.29%) | 2.280 [1.674; 3.105] | <0.0001 |

Supplementary Table 9. Proportion of patients with resolution of pessimistic thoughts based on MADRS (NRI)

Full methodology reported in **Appendix 1**. MADRS: Montgomery-Åsberg Depression Rating Scale; NRI: non-responder imputation; NS: nasal spray; SNRI: serotonin norepinephrine reuptake inhibitor; SSRI: selective serotonin reuptake inhibitor; XR: extended release.

| **Week** | **Esketamine NS + SSRI/SNRI (N=336)** | **Quetiapine XR + SSRI/SNRI (N=340)** | **Odds Ratio** | **p-value** |
| --- | --- | --- | --- | --- |
| 1 | 267 (79.46%) | 259 (76.18%) | 1.219 [0.845; 1.758] | 0.2894 |
| 2 | 282 (83.93%) | 258 (75.88%) | 1.673 [1.139; 2.459] | 0.0085 |
| 4 | 286 (85.12%) | 252 (74.12%) | 2.009 [1.363; 2.961] | 0.0004 |
| 6 | 281 (83.63%) | 253 (74.41%) | 1.763 [1.207; 2.574] | 0.0032 |
| 8 | 272 (80.95%) | 244 (71.76%) | 1.673 [1.167; 2.400] | 0.0050 |
| 10 | 269 (80.06%) | 230 (67.65%) | 1.924 [1.355; 2.732] | 0.0002 |
| 12 | 269 (80.06%) | 226 (66.47%) | 2.028 [1.431; 2.876] | <0.0001 |
| 14 | 265 (78.87%) | 216 (63.53%) | 2.154 [1.529; 3.035] | <0.0001 |
| 16 | 260 (77.38%) | 217 (63.82%) | 1.960 [1.396; 2.752] | <0.0001 |
| 18 | 263 (78.27%) | 209 (61.47%) | 2.278 [1.622; 3.199] | <0.0001 |
| 20 | 254 (75.60%) | 210 (61.76%) | 1.939 [1.390; 2.705] | <0.0001 |
| 22 | 250 (74.40%) | 210 (61.76%) | 1.821 [1.309; 2.534] | 0.0004 |
| 24 | 248 (73.81%) | 208 (61.18%) | 1.806 [1.301; 2.508] | 0.0004 |
| 26 | 245 (72.92%) | 206 (60.59%) | 1.770 [1.277; 2.452] | 0.0006 |
| 28 | 248 (73.81%) | 203 (59.71%) | 1.918 [1.383; 2.660] | <0.0001 |
| 30 | 245 (72.92%) | 200 (58.82%) | 1.893 [1.369; 2.617] | 0.0001 |
| 32 | 249 (74.11%) | 198 (58.24%) | 2.068 [1.492; 2.866] | <0.0001 |

Supplementary Table 10. Proportion of patients with resolution of suicidal thoughts based on MADRS (NRI)

Full methodology reported in **Appendix 1**. MADRS: Montgomery-Åsberg Depression Rating Scale; NRI: non-responder imputation; NS: nasal spray; SNRI: serotonin norepinephrine reuptake inhibitor; SSRI: selective serotonin reuptake inhibitor; XR: extended release.

**Supplementary Figure 1. ESCAPE-TRD study design**


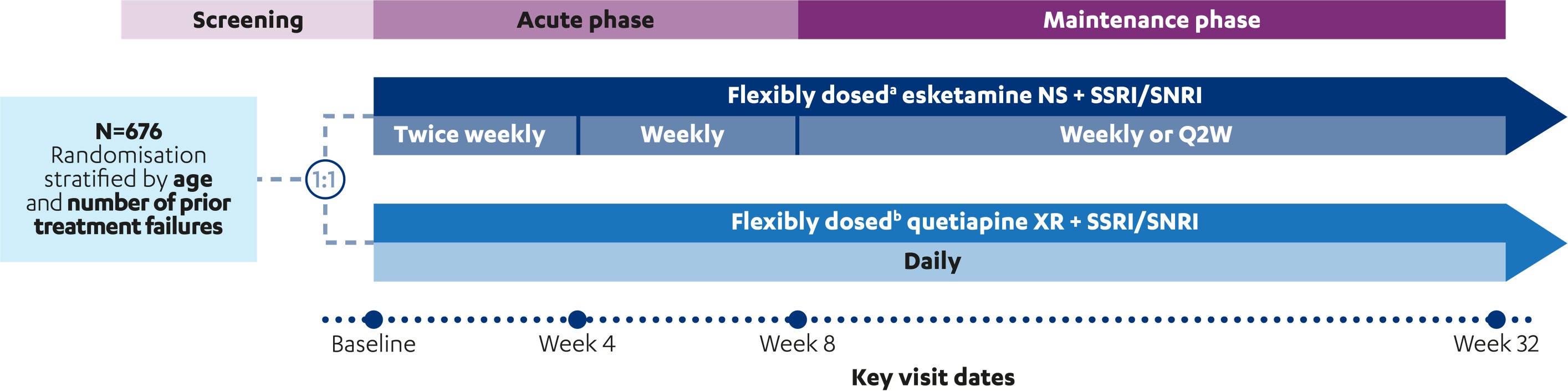


ESCAPE-TRD (NCT04338321) was a randomised, open-label, MADRS rater-blinded, active-controlled phase IIIb study that evaluated the efficacy and safety of esketamine NS versus quetiapine XR in combination with an ongoing SSRI/SNRI, in patients with TRD. [a] Esketamine NS was dosed twice weekly (56 mg on Day 1, 56/84 mg from Day 4) from Week 1–4, weekly (56/84 mg) from Week 5–8 and weekly or Q2W (56/84 mg) from Week 9–32, all in addition to an ongoing SSRI/SNRI that elicited non-response at baseline; [b] Quetiapine XR was flexibly dosed and administered daily, starting at 50 mg on Days 1–2, 150 mg/day on Days 3–4 and 300 mg/day from Day 5 onwards, all in addition to an ongoing SSRI/SNRI that elicited non-response at baseline. NS: nasal spray; Q2W: every two weeks; SNRI: serotonin norepinephrine reuptake inhibitor; SSRI: selective serotonin reuptake inhibitor; TRD: treatment resistant depression; XR: extended release.


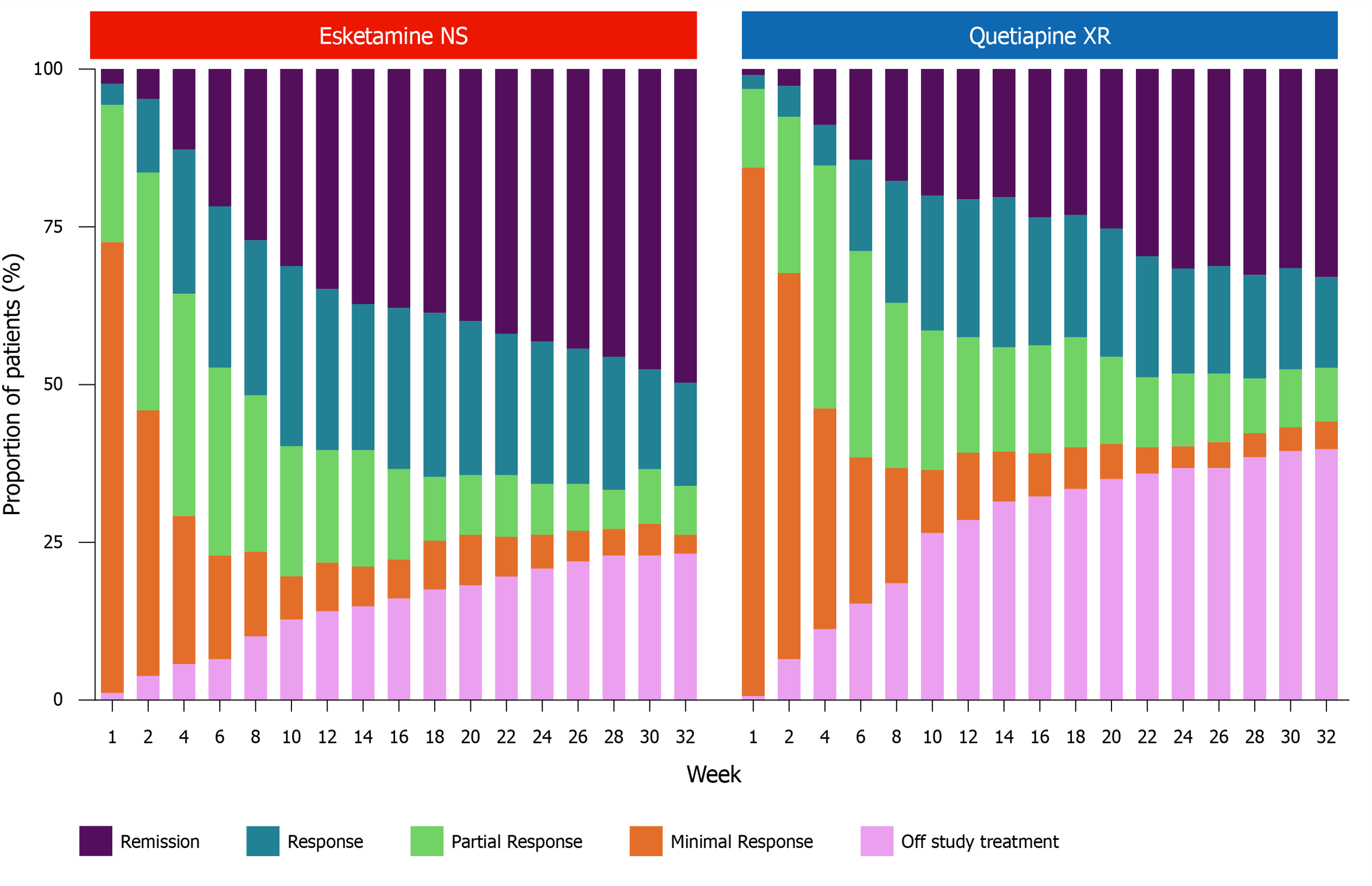
Supplementary Figure 2. Esketamine NS and quetiapine XR patient outcome trajectories

Full analysis set in ESCAPE-TRD; N=676; esketamine NS: n=336; quetiapine XR: n=340. For patients who remained on treatment, missing visits were imputed using LOCF. Remission was defined as MADRS total score ≤10. Other outcomes were defined as follows, though all are considered “without remission” (MADRS total score >10): response was defined as a ≥50% reduction from baseline in MADRS, partial response as a 25–<50% reduction from baseline in MADRS and minimal-response as a <25% reduction from baseline in MADRS. MADRS: Montgomery-Åsberg Depression Rating Scale; LOCF: last observation carried forward; NS: nasal spray; XR: extended release.Supplementary Figure 3. Outcome stability with esketamine NS following partial response at Week 4 (A), partial response at
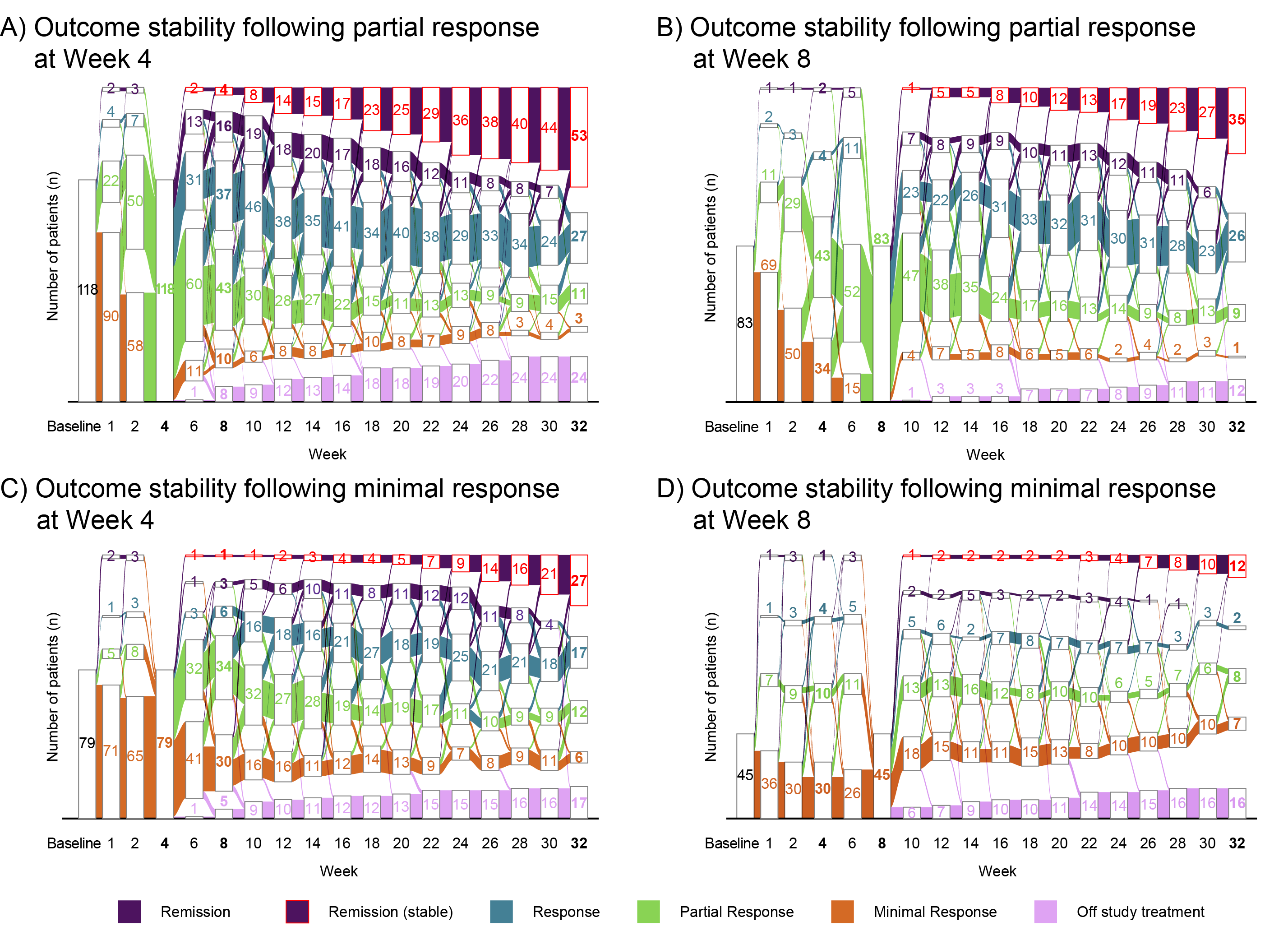
Week 8 (B), minimal response at Week 4 (C) and minimal response at Week 8 (D) [LOCF]

Full analysis set in ESCAPE-TRD; N=676; esketamine NS: n=336. Patient trajectories between outcomes with esketamine NS treatment during ESCAPE-TRD. Remission was defined as MADRS total score ≤10. LOCF was applied for patients who remained on treatment with missing MADRS data at the given visit. Other outcomes were defined as follows, though all are considered “without remission” (MADRS total score >10): response was defined as a ≥50% reduction from baseline in MADRS, partial response as a 25–<50% reduction from baseline in MADRS and minimal response as a <25% reduction from baseline in MADRS. Remission with stable outcome (highlighted in red) was defined for patients in remission (MADRS total score ≤10) at a given week who remained in remission for all subsequent weeks (using LOCF) until Week 32. LOCF: last observation carried forward; NS: nasal spray; MADRS: Montgomery-Åsberg Depression Rating Scale.
